# Synthesis and Influence of 3-Amino Benzoxaboroles Structure on Their Activity against *Candida albicans*

**DOI:** 10.3390/molecules25245999

**Published:** 2020-12-18

**Authors:** Dorota Wieczorek, Ewa Kaczorowska, Marta Wiśniewska, Izabela D. Madura, Magdalena Leśniak, Jacek Lipok, Agnieszka Adamczyk-Woźniak

**Affiliations:** 1 Faculty of Chemistry, University of Opole, Oleska 48, 45-052 Opole, Poland; dorwieczorek@uni.opole.pl (D.W.); jacek.lipok@uni.opole.pl (J.L.); 2 Faculty of Chemistry, Warsaw University of Technology, Noakowskiego 3, 00-664 Warsaw, Poland; ewak@ch.pw.edu.pl (E.K.); marta.wisniewska955@gmail.com (M.W.); izabela@ch.pw.edu.pl (I.D.M.); lesniakmagda@onet.pl (M.L.)

**Keywords:** benzoxaboroles, Kerydin, antifungal, piperazine, formyl, *Candida albicans*

## Abstract

Benzoxaboroles emerged recently as molecules of high medicinal potential with Kerydin^®^ (Tavaborole) and Eucrisa^®^ (Crisaborole) currently in clinical practice as antifungal and anti-inflammatory drugs, respectively. Over a dozen of 3-amino benzoxaboroles, including Tavaborole’s derivatives, have been synthetized and characterized in terms of their activity against *Candida albicans* as a model pathogenic fungus. The studied compounds broaden considerably the structural diversity of reported benzoxaboroles, enabling determination of the influence of the introduction of a heterocyclic amine, a fluorine substituent as well as the formyl group on antifungal activity of those compounds. The determined zones of the growth inhibition of examined microorganism indicate high diffusion of majority of the studied compounds within the applied media as well as their reasonable activity. The Minimum Inhibitory Concentration (MIC) values show that the introduction of an amine substituent in position “3” of the benzoxaborole heterocyclic ring results in a considerable drop in activity in comparison with Tavaborole (AN2690) as well as unsubstituted benzoxaborole (AN2679). In all studied cases the presence of a fluorine substituent at position *para* to the boron atom results in lower MIC values (higher activity). Interestingly, introduction of a fluorine substituent in the more distant piperazine phenyl ring does not influence MIC values. As determined by X-ray studies, introduction of a formyl group in proximity of the boron atom results in a considerable change of the boronic group geometry. The presence of a formyl group next to the benzoxaborole unit is also detrimental for activity against *Candida albicans*.

## 1. Introduction

One of the applicable features of benzoxaboroles is their antifungal activity [1]. Over the last few decades, the frequency of infections caused by fungi has increased significantly. Most patients are diagnosed with superficial (mucosal or cutaneous) mycosis. However, invasive opportunistic mycoses associated with the infection of various internal organs such as liver, lungs, spleen, or brain are also often diagnosed. The most exposed group of people for such a type of infections are immune-compromised individuals including solid organ and blood or bone marrow transplant recipients as well as HIV positive or cancer patients [2,3]. The most common causes of mycoses are *Candida* spp. There are at least over a dozen *Candida* species that cause human infection but more than 90% of invasive diseases is caused by the five most common pathogens: *C. albicans*, *C. glabrata*, *C. tropicalis*, *C. parapsilosis*, and *C. krusei*. Among those infection *C. albicans* remains the most common one [4]. Despite *Candida* is the leading reason of the opportunistic mycoses, there is a limited number of antifungal agents available for the therapy [5].

**Figure 1:**
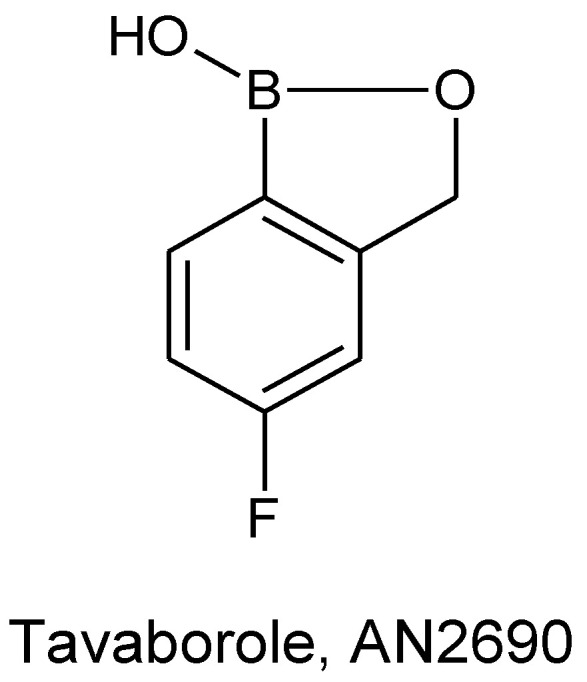
The structure of Tavaborole (AN2690), active ingredient of Keridin–antifungal drug approved by FDA in 2014 for the treatment of onychomycosis.

Tavaborole (AN2690, Figure 1) is a small-molecule approved by the American Food and Drug Administration (FDA) in 2014 for the treatment of onychomycosis caused by dermatophyte fungi [6,7,8,9,10,11,12].

A relatively low molecular weight of Tavaborole results in high penetration into the nailplate [13]. This may compensate its considerably lower antifungal activity determined in in vitro studies in comparison with three different drugs such as: Raconazole, terbinafine, and fluconazole [14]. Tavaborole acts by blocking the fungus’s ability to produce proteins in a highly specific way. It disrupts the catalytic action of yeast cytoplasmic enzyme called leucyl-tRNA synthetase involved in translation process [15]. This mechanism named as the oxaborole tRNA trapping mechanism (OBORT mechanism) is exclusive for benzoxaborole compounds, while other popular antifungal drugs such as fluconazole, itraconazole, terbinafine, efinaconazole and amorolfine act by inhibiting fungal cell wall synthesis or by weakening microbial metabolism [16]. The synthesis of analogs of chemical compounds with proven therapeutic effect is a commonly used method in the design of new drugs. It allows to increase the efficiency of analogs in relation to the lead as well as to reduce the costs of the entire drug development process [17,18].

**Figure 2:**
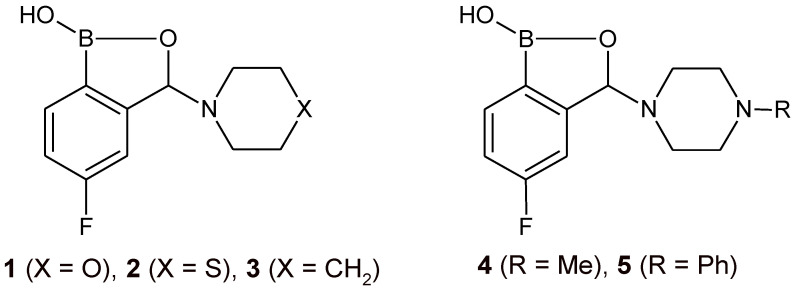
Structures of studied Tavaborole’s derivatives (**1**–**5**).

**Figure 3:**
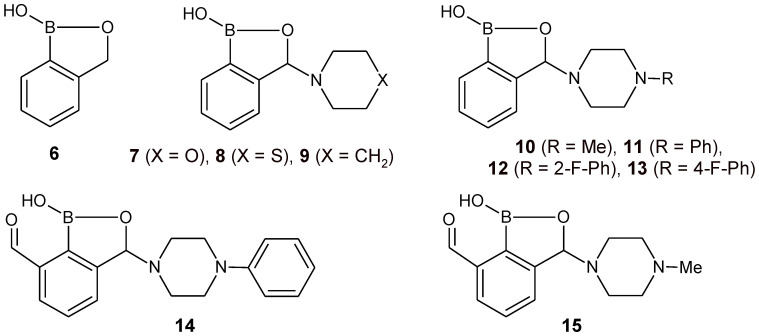
Structures of unsubstituted benzoxaborole (**6**) and its studied derivatives (**7**–**15**).

The current study evaluates analogs of Tavaborole (**1**–**5**, Figure 2) as well as unsubstituted benzoxaborole (**6** (AN2679), Figure 3) and its analogs (**7**–**15**, Figure 3) as potential agents against *Candida albicans*. Although several molecules combining benzoxaborole and heterocyclic amine structural motives have displayed promising antifungal activity [19,20,21], none of them has been previously studied as agents against *Candida albicans*. At the same time, some of the very recently described piperazine bis(benzoxaboroles) displayed reasonable activity against that microorganism [22]. The studied compounds broaden considerably the structural diversity of reported benzoxaboroles, enabling determination of the influence of the presence of a heterocyclic amine, a fluorine substituent as well as the formyl group on antifungal activity of those compounds.

## 2. Results and Discussion

### 2.1. Synthesis and Structural Studies

**Scheme 1:**
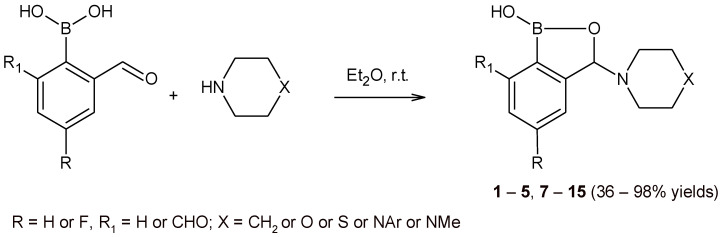
Synthetic procedure for the studied 3-aminobenzoxaboroles.

Most of the studied 3-amino-benzoxaboroles are novel compounds (**2**–**5**, **10**, **11**, **14** and **15**) and have been obtained according to a very convenient procedure by mixing the starting (2-formylphenyl)boronic acid with the corresponding amine at room temperature in diethyl ether (Scheme 1).

Low solubility of several 3-aminobenzoxaboroles (**4**, **5**, **10**, **11**, **14** and **15**) in diethyl ether caused their precipitation from a reaction mixture, driving the reaction to completion as well as enabling straightforward isolation and purification of the products. In some cases, subsequent crystallization was used to purify the products (see experimental section). Contrary to some of the previously reported synthetic procedures for 3-aminobenzoxaboroles [20,23,24] and similarly as in the synthesis of bis(benzoxaboroles) [25] no drying agent was used. Most of the compounds have been fully characterized by NMR spectroscopy (^1^H, ^13^C, ^11^B NMR spectra). If applicable, ^19^F NMR spectra have also been provided. The unsubstituted benzoxaborole (**6**) was obtained by reduction of the corresponding (2-formylphenyl)boronic acid as it was previously reported [26,27]. Compounds **7**–**9** [24], **12** and **13** [20] were obtained as reported previously. Most of the samples used for microbiological studies gave satisfactory results of elemental analysis, which confirms their homogeneity and purity. However, in case of compounds **2**, **3**, **7**–**9** results of elemental analysis as well as ^1^H NMR spectra showed contamination of the products with diethyl ether and/or the starting amine. Those impurities could not be removed neither under high vacuum nor by washing with hexane, which suggests co-crystallization of those compounds with amine or diethyl ether. It is worth noting that the presence of diethyl ether was previously detected in crystals of **13** by X-ray [20]. All the studied molecules are stable in the solid state at room temperature. On heating however, most of them decompose prior or instead of melting (see experimental section for decomposition temperatures).

**Figure 4:**
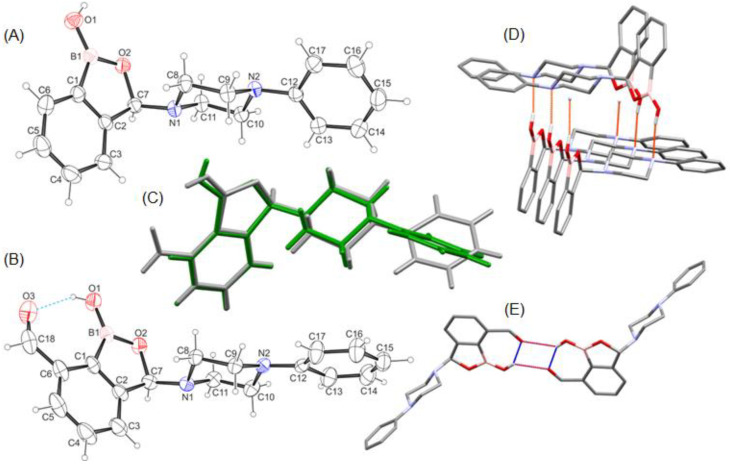
(**A**,**B**) Ortep drawings [30] of **11** and **14** molecules with atoms numbering scheme, (**C**) Molecules overlay showing the difference in phenyl ring orientation; (**D**) 1-D supramolecular chain formed by molecules of **11** joined by O-H…N hydrogen bonds (in orange); (**E**) Hydrogen-bonded dimer of **14** (intra- and intermolecular H-bonds are shown as blue and red lines, respectively. In (**D**,**E**) hydrogen atoms not involved in hydrogen bonds are omitted for clarity.

Most of the so far reported benzoxaboroles, including AN2690 [26], **6** [27], **7** [28] and **8** [23] form dimers in the solid state. This does not account for the recently reported phenylpiperazine derivatives **12** and **13**, both displaying pretty unusual structural patterns for benzoxaboroles [20]. In search for a structure-activity relationship, molecular structures of novel phenylpiperazine derivatives **11** and **14** were determined in crystalline state. Similarly to **12** and **13**, compounds **11** and **14** crystallize as an equimolar mixture of anantiomers (racemate). Molecular structures of **11** and **14** are shown in Figure 4A,B. Both crystals exhibit symmetry of *P*2_1_/*n* centrosymmetric space group of the monoclinic system and in each case one molecule is present in the asymmetric unit (see Table S1, Supplementary Materials). While compounds **11** and **12** are isostructural, the introduction of the formyl group in **14** changes the geometry of the boron vicinity considerably. In **11** and **14** molecules the boron atom shows a trigonal coordination and the B–O and B–C bond lengths are comparable for both molecules (see Table S2, Supplementary Materials). However, in the case of **14** the insertion of the formyl substituent in the *ortho* position relative to the boronic moiety causes the significant O1–B1–O2 angle narrowing (from 123.1(1) in **11** to 118.4(2)° in **14**) with simultaneous O1–B1–C1 angle broadening (from 128.3(1) in **11** to 133.5(2)° in **14**), whereas the five-membered ring inner O2–B1–C1 angle is retained. This is following the findings by Czerwińska et al. [29] associating the strains in the boron coordination sphere with the position of the hydrogen atom in *syn* (**11**) or *anti* position (**14**). In the latter such a conformation may be imposed by the presence of a relatively strong intramolecular O1–H…O3 hydrogen bond (H…O and O…O distances equal 2.15(2) and 2.867(2) Å, respectively; O–H…O angle equals 147(2)°), leading to a formation of a six-membered H-bonded ring, essentially flat and coplanar with the benzoxaborole unit (deviation from a plane defined by all atoms except the phenylpiperazine substituent is only 0.019(2) Å).

Interestingly, the OH donor in **14** is also engaged in the formation of a weak intermolecular hydrogen bonding with O3 formyl oxygen atom of the molecule related by the inversion center (H…O and O…O distances equal 2.38(2) and 3.033(2) Å, respectively; O–H…O angle is 137(2)°). As a result, a new type of an H-bonded dimer is created (Figure 4E), not previously observed in structurally characterized benzoxaboroles. Whereas in **11** a relatively strong O1-H…N2 intermolecular hydrogen bond is observed (H…N and O…N distances equal 2.13(2) and 2.962(1) Å, respectively; O–H…N angle is 171(1)°), leading to the formation of an infinite chain along 2_1_ screw axis (Figure 4D). It is noteworthy that the same motif was previously observed in the isostructural 3-[4-(2-fluorophenyl)piperazin-1-yl]-2,1-benzoxaborol-1(3H)-ol (**12**) [20]. The other difference between **11** and **14** molecules is a twist of the phenyl ring with respect to the piperazine ring (Figure 4C), arising probably from packing factors as in both cases these rings are not involved in any significant intermolecular interactions.

### 2.2. Studies of Activity against Candida albicans

**Figure 5:**
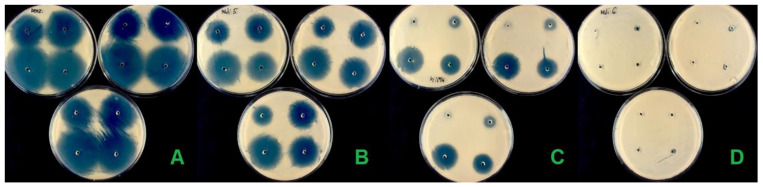
Three repetitions of the agar diffusion method tests for compounds: **6** (**A**), **5** (**B**), **11** (**C**) and **14** (**D**).

The antifungal activity of all the studied benzoxaboroles (**1**–**15**) against *C. albicans* has been evaluated by the diffusion agar method as well as by determination of the MIC values. Results were compared to that of Tavaborole (AN2690, Keridin) reported previously and carried out under strictly the same protocol [31]. Additionally, amphotericin B was used as a positive standard and DMSO as a negative probe. Antifungal activity was evaluated by measuring the diameter of the clear zone surrounding the holes. The results are shown in Table 1. Figure 5 shows pictures of agar diffusion method for chosen compounds (**5**, **6**, **11** and **14**) in three repetitions.

The agar diffusion tests show that most of the investigated benzoxaboroles display higher diameter of the inhibition zone in comparison with a standard amphotericin B (9 mm at 100 μg).

The results indicate high diffusion of the investigated benzoxaboroles with the applied solid medium as well as their reasonable antifungal activity. The highest values are observed for the unsubstituted benzoxaborole (**6**) with the diameter of 44 mm at 100 μg (Figure 5A), which is smaller yet comparable to that recently reported for AN2690 (54 mm at 100 μg) [31]. In case of the investigated 3-substituted benzoxaboroles **1**–**5** and **7**–**13** the diameters of the zones of totally inhibited growth of *Candida albicans* are considerably lower and range from 21 mm for **11** to 34 mm for **4** at 100 μg. Two of the 3-aminobenzoxaboroles studied (**14** and **15**), both containing formyl group in boron atom proximity, reveal no inhibition of the growth of *Candida albicans* at applied conditions (Figure 5D for **14**). Similar results have been previously reported for the (2,6-diformylphenyl)boronic acid [32].

The determined values of the Minimal Inhibition Concentration (MIC) allow to arrange the investigated compounds **1**–**15** with respect to their activity against *Candida albicans*. The most potent one is the unsubstituted benzoxaborole (**6**) with MIC value of 8 μg/mL, which is however four times higher than that of Tavaborole (AN2690). Introduction of the amine substituent at position “3” of the heterocyclic ring results in a considerable drop of the activity. The trend is similar in case of analogues of Tavaborole (**1**–**5**) as well as compound **6** and its derivatives (**7**–**15**).

In both cases the piperazine derivatives–compounds **4**, **5** and **10**–**13** display higher activity in comparison of morpholine, thiomorpholine and piperidine derivatives (**1**–**3** and **7**–**9** respectively). The MIC values range from about 31 to 62 μg/mL for the 3-amino analogues of Tavaborole (**1**–**5**) and from about 62 to 125 μg/mL for analogues of **6**, which contain no fluorine substituent at position *para* to the boron atom. These results show that the presence of a fluorine atom at position *para* to the boronic group enhances antifungal activity. Interestingly, no such effect was observed for introduction of the fluorine atom in a more distant part of the molecule i.e., in the phenyl piperazine ring in compounds **12** and **13**.

## 3. Conclusions

The structure of reported benzoxaboroles influences considerably their activity against *Candida albicans*. The unsubstituted benzoxaborole (**6**) displays the highest activity amongst all the investigated compounds. In all of the studied cases, introduction of the amino substituent in position “3” of the heterocyclic ring results in a significant drop in activity. The unfavorable effect is higher for simple heterocyclic amines derivatives such as morpholine, thiomorpholine and piperidine than for the piperazine derivatives that still display a reasonable activity. The other structural factor considerably influencing performance of investigated benzoxaboroles is the presence of a fluorine substituent at position *para* to the boron atom, which results in lower MIC values (higher activity). This effect is the most profound for unsubstituted benzoxaborole compared with Tavaborole, where the lack of the fluorine atom results in four times higher MIC value (lower activity) for the unsubstituted benzoxaborole in comparison with the known drug. In case of investigated 3-amino-benzoxaboroles the effect is smaller, yet noticeable. All derivatives containing fluorine substituent at position *para* to the boron atom display twice lower MIC values comparing to derivatives containing no such a substituent. Interestingly, no influence on activity was observed for derivatives containing fluorine atom in the more distant phenylpiperazine substituent. It is worth noticing that the presence of the formyl group in the boron vicinity influences the geometry of the boronic group considerably and is detrimental for the activity against *Candida albicans*.

## 4. Experimental Section

### 4.1. Materials and Methods

All reactions were performed under air, using undried glassware and undistilled solvents. Reagents and solvents were obtained from commercial sources (Sigma-Aldrich, St. Louis, MO, USA, POCH, ChemPur) and used without further purification. Deuterated solvents were obtained from Armar Chemicals and used as received. The reported yields correspond to isolated products.

NMR spectra were recorded on a Bruker Avance 300 MHz or Varian NMR System 500 MHz spectrometers in CDCl_3_ or (CD_3_)_2_CO at room temperature. Chemical shifts are reported in parts per million (ppm), relative to the residual undeuterated solvent signal as an internal standard. All signals are reported as follows: chemical shift (multiplicity, number of corresponding nuclei), using the following abberviations: s = singlet, m = multiplet, d = doublet, dd = doublet of doublets, t = triplet, br = broad signal.

Elemental analyses were obtained using a Perkin Elmer 2400 apparatus.

All the Yeast Peptone Dextrose (YPD) medium components as well as Amphotericin B, were obtained from Sigma–Aldrich. DMSO was purchased from POCh. *Candida albicans* LOCK 001 was obtained from the collection of microorganisms of Institute of Fermentation Technology and Microbiology (University of Technology, Łódź, Poland).

Studies of the activity against *C. albicans* by the diffusion agar method: 0.5 mL of inoculum containing 10^6^–10^7^ fungal cells were spread on the surface of the solidified yeast extract peptone dextrose (YPD) medium and allowed to dry. Portions of 100, 50, 25 and 10 μg of the tested compounds dissolved in DMSO were placed in 2 mm diameter holes, which were cut into the media. The duration of incubation of the fungi was established as 48 h at 27 °C. Each experiment, including control, was carried out in three repetitions.

Determination of MIC values: A 24-h-old culture of *C. albicans* was diluted with a fresh YPD medium to obtain optical density of 0.02 at 600 nm (OD600). The investigated compounds were dissolved in DMSO and placed in the initial culture of yeast (2 mL), ensuring final concentration ranging from 0.9 to 500 μg mL^−1^). Cells were incubated for 24 h at 25 °C on a rotary shaker (60 rpm). After that time, the OD600 measurements were performed using a spectrophotometer (UV-2601, Rayleigh). The MIC endpoints defined as the lowest concentration at which no visible growth of yeast was observed were read visually following 24 h of incubation. Each experiment (control or compound-containing medium) was repeated three times.

The single crystal X-ray experiments were conducted at room temperature on the Gemini A Ultra Diffractometer (Rigaku Oxford Diffraction) using graphite monochromated Mo/Kα radiation (λ = 0.71073 Å). Data collection and reduction were performed in the CrysAlis^Pro^ program [33]. The empirical absorption corrections using spherical harmonics, implemented in multi-scan scaling algorithm were applied. OLEX-2 suite [34] with implemented ShelXT [35] structure solution program using Intrinsic Phasing and the ShelXL [36] refinement package (the full-matrix least-squares technique) were used to solve and refine the structures. All non-hydrogen atoms were refined with anisotropic temperature factors. The carbon hydrogen atoms were placed in calculated positions while those bonded with oxygen atoms were refined freely. For all hydrogen atoms fixed isotropic thermal parameters (*U*_iso_(H) = 1.2 × [*U*_eq_(C) or *U*_eq_(O)] were used. The final refinement indices for the observed data converged to *R*_1_ = 4.39% and *wR*_2_ = 9.79% for **11** and *R*_1_ = 5.73% and *wR*_2_ = 12.34% for **14** (see Table S1 in the Supplementary Materials). Selected geometrical parameters are collected in Table S2 in the Supplementary Materials file. Molecular and packing diagrams were generated using MERCURY [37] and ORTEP-3 for Windows [30] programs. CCDC 1998837-1998838 contain the supplementary crystallographic data for this paper. The data can be obtained free of charge from The Cambridge Crystallographic Data Centre via www.ccdc.cam.ac.uk/structures.

### 4.2. Synthetic Procedures and Characterization Data

5-Fluoro-3-morpholin-4-yl-2,1-benzoxaborol-1(3H)-ol (**1**)

(4-fluoro-2-formylphenyl)boronic acid (0.235 g, 1.39 mmol) and diethyl ether (10 mL) were placed in a glass vial and mixed until dissolved. Morpholine (0.122 mL, 1.39 mmol) was added to the solution and mixed on a magnetic stirred for several minutes. The reaction mixture was left in the open vessel for one day to self-evaporate most of the Et_2_O (longer time in the open vessel did not result in the evaporation of more solvent). On the next day, the glassy body obtained was subjected to evaporation in a vacuum to remove remnant volatiles resulting in 0.322 g of the solid, yield: 97%.

^1^H NMR (300 MHz, Acetone) δ 8.11 (s, br, 1H, B-OH), 7.73 (dd, *J* = 8.0, 5.6 Hz, 1H), 7.28–7.00 (m, 2H), 5.80 (s, 1H), 3.62–3.99 (m, 4H), 2.72–2.59 (m, 2H), 2.58–2.43 (m, 2H). ^13^C NMR (75 MHz, Acetone) δ 166.1 (d, ^1^*J*c_F_ = 247.7 Hz), 156.7 (d, ^3^*J*c_F_ = 8.1 Hz), 133.1 (d, ^3^*J*c_F_ = 9.0 Hz), 116.7 (d, ^2^*J*c_F_ = 22.3 Hz), 110.5 (d, ^2^*J*c_F_ = 22.0 Hz), 96.6 (d, ^3^*J*c_F_ = 3.5 Hz), 67.50, 48.05. ^19^F NMR (282 MHz, Acetone) δ −111.12 (m). ^11^B NMR (96 MHz, Acetone) δ 31 (s). EA calculated for C_11_H_13_BFNO_3_: C(55.74%), H(5.53%), N(5.91%), determined: C(56.29%), H(5.87%), N(7.00%). m.p. = 65–105 °C (decomposition).

5-Fluoro-3-thiomorpholin-4-yl-2,1-benzoxaborol-1(3H)-ol (**2**)

(4-fluoro-2-formylphenyl)boronic acid (0.234 g, 1.39 mmol) and diethyl ether (10 mL) were placed in a glass vial and mixed until dissolved. Tiomorpholine (0.139 mL, 1.39 mmol) was added to the solution and mixed on a magnetic stirred for several minutes. The reaction mixture was left in the open vessel for one day to self-evaporate most of the Et_2_O (longer time in the open vessel did not result in the evaporation of more solvent). On the next day, the glassy body obtained was subjected to evaporation in a vacuum to remove remnant volatiles resulting in 0.342 g of the solid, yield: 97%.

^1^H NMR (300 MHz, CDCl_3_): δ (ppm) = 7.70–7.67 (m, 1H, Ar), 7.14–7.08 (m, 2H, Ar), 5.79 (s, 1H, CH), 3.22–3.19 (m, 2H, CH_2_N), 2.93–2.83(m, 2H, CH_2_N), 2.68–2.64 (m, 4H, (CH_2_)_2_S). ^19^F NMR (282 MHz, CDCl_3_): δ (ppm) = −108.8 (s, br). ^11^B NMR (96 MHz, CDCl_3_): δ (ppm) = 31. EA calculated for C_11_H_13_BFNO_2_S: C(52.20%), H(5.18%), N(5.53%), determined: C(52.28%), H(5.40%), N(5.42%). m.p. = 70–85 °C (decomposition).

5-Fluoro-3-piperidin-1-yl-2,1-benzoxaborol-1(3H)-ol (**3**)

(4-Fluoro-2-formylphenyl)boronic acid (0.238 g, 1.42 mmol) and diethyl ether (10 mL) were placed in a glass vial and mixed until dissolved. Piperidine (0.140 mL, 1.42 mmol) was added to the solution and mixed on a magnetic stirred for several minutes. The reaction mixture was left in the open vessel for one day to self-evaporate most of the Et_2_O (longer time in the open vessel did not result in the evaporation of more solvent). On the next day, the glassy body obtained was subjected to evaporation in a vacuum to remove remnant volatiles resulting in 0.313 g of the solid, yield: 94%.

^1^H NMR (300 MHz, CDCl_3_): δ (ppm) = 7.59 (s, br, 1H, Ar), 7.11–7.08 (m, 2H, Ar), 5.77 (s, br, 1H, CH) 2.94, (s, br, 2H, CH_2_), 2.59 (s, br, 2H), 1.63–1.47), 6H, (CH_2_)_3_). ^19^F NMR (282 MHz, CDCl_3_): δ (ppm) = −109.6. ^11^B NMR (96 MHz, CDCl_3_): δ (ppm) = 30. EA calculated for C_12_H_15_BFNO_2_: C(61.31%), H(6.43%), N(5.96%); determined: C(63.44%), H(6.97%), N(7.04%). m.p. = 67–75 °C (decomposition).

5-Fluoro-3-(4-methylpiperazin-1-yl)-2,1-benzoxaborol-1(3H)-ol (**4**)

A solution of (4-fluoro-2-formylphenyl)boronic acid (1.5 g; 9 mmol) in diethyl ether (85 mL) was stirred in a 250 mL flask at room temperature. Next, 1-methylpiperazine (0.9 g; 1.0 mL; 9 mmol) was added during 3 min resulting immediately in precipitation of a white solid. It was filtered, washed with diethyl ether (2 × 3 mL) and dried to give the title compound as white solid. Yield: 1.48 g; 6 mmol; 65%;

^1^H NMR (500 MHz, CDCl_3_) δ 7.66 (dd, *J* = 8.0, 5.6 Hz, 1H, Ar), 7.16–6.95 (m, 2H, Ar), 5.78 (s, 1H, CH), 3.20–2.00 (m, br, 8H, CH_2_N), 2.32 (s, 3H, CH_3_). ^13^C NMR (126 MHz, CDCl_3_) *δ* 165.3 (d, ^1^*J*_CF_ = 248.9 Hz), 155.6 (d, ^3^*J*_CF_ = 8.3 Hz), 132.0 (d, ^3^*J*_CF_ = 8.9 Hz), 116.0 (d, ^2^*J*_CF_ = 21.9 Hz), 109.8 (d, ^2^*J*_CF_ = 21.7 Hz), 95.8 (d, ^4^*J*_CF_ = 3.1 Hz), 55.2 (s), 45.8 (s). ^11^B NMR (75 MHz, CDCl_3_): *δ* 30 (s, br). ^19^F NMR (282 MHz, CDCl_3_): *δ* −110.09–110.18 (m). EA calculated for C_12_H_16_N_2_BO_2_F: C(57.63%), H(6.45%), N(11.20%); determined: C(57.72%), H(6.51%), N(11.12%). m.p. = 166–169 °C (decomposition, browning).

5-Fluoro-3-(4-phenylpiperazin-1-yl)-2,1-benzoxaborol-1(3H)-ol (**5**)

A solution of (4-fluoro-2-formylphenyl)boronic acid (1.5 g; 9 mmol) in diethyl ether (90 mL) was stirred in a 250 mL flask at room temperature. Next, 1-phenylpiperazine (1.4 g; 1.4 mL; 9 mmol) was added during 3 min resulting in precipitation of a white solid after 24 h. It was filtered, washed with diethyl ether (2 × 3 mL) and dried to give the title compound as a white solid. Yield: 0.99 g; 3 mmol; 36%;

^1^H NMR (300 MHz, CDCl_3_) δ 7.69 (dd, *J* = 8.0, 5.6 Hz, 1H, Ar), 7.31–7.22 (m, 2H, Ar), 7.19–7.06 (m, 2H, Ar), 6.93 (d, *J* = 8.0 Hz, 2H, Ar), 6.87 (t, *J* = 7.3 Hz, 1H, Ar), 5.92 (s, 1H), 3.18 (t, *J* = 5.0 Hz, 4H), 3.01–2.64 (m, 4H). ^13^C NMR (126 MHz, CDCl_3_) δ 165.5 (d, ^1^*J*_CF_ = 249.4 Hz), 151.2, 132.2 (d, ^3^*J*_CF_ = 9.6 Hz), 129.3, 129.1, 119.9, 116.9, 116.4 (d, ^2^*J*_CF_ = 22.2 Hz), 116.3, 110.1 (d, ^2^*J*_CF_ = 22.0 Hz), 96.7, 49.4, 46.7. ^11^B NMR (96 MHz, CDCl_3_): *δ* 30. ^19^F NMR (282 MHz, CDCl_3_): *δ* −108.4 (m). EA calculated for C_17_H_18_N_2_B0_2_F: C(65.41%), H(5.81%), N(8.97%); determined: C(65.60%), H(5.90%), N(9.11%). m.p. = 160–163 °C (decomposition).

2,1-Benzoxaborol-1(3*H*)-ol (**6**)

^1^H NMR (400 MHz, Acetone) δ 8.13 (s, 1H, B(OH)), 7.73 (d, *J* = 7.3 Hz, 1H, Ar), 7.49–7.43 (m, 1H, Ar), 7.42–7.38 (m, 1H, Ar), 7.33 (td, *J* = 7.3, 1.0 Hz, 1H, Ar), 5.01 (s, 2H).

3-Morpholin-4-yl-2,1-benzoxaborol-1(3H)-ol (**7**)

(2-formylphenyl)boronic acid (0.252 g, 1.68 mmol) and diethyl ether (10 mL) were placed in a glass vial and mixed until dissolved. Morpholine (0.147 mL, 1.68 mmol) was added to the solution and mixed on a magnetic stirred for several minutes. The reaction mixture was left in the open vessel for one day to self-evaporate most of the Et_2_O (longer time in the open vessel did not result in the evaporation of more solvent). On the next day, the glassy body obtained was subjected to evaporation in a vacuum to remove remnant volatiles resulting in 0.350 g of the solid, yield: 95%.

^1^H NMR (300 MHz, CDCl_3_) δ 7.69 (d, *J* = 6.6 Hz, 1H, Ar), 7.54–7.32 (m, 3H, Ar), 5.85 (s, 1H, CH), 3.71–3.68 (m, 4H), 2.73–2.61 (m, 4H). ^11^B NMR (96 MHz, CDCl_3_): δ (ppm) 31. EA calculated for C_11_H_14_BNO_3_: C(60.32%), H(6.44%), N(6.39%); determined: C(60.27%), H(6.42%), N(6.37%). m.p. = 63–80 °C (decomposition).

3-Thiomorpholin-4-yl-2,1-benzoxaborol-1(3H)-ol (**8**)

(2-formylphenyl)boronic acid (0.202 g, 1.35 mmol) and diethyl ether (10 mL) were placed in a glass vial and mixed until dissolved. Thiomorpholine (0.135 mL, 1.35 mmol) was added to the solution and mixed on a magnetic stirred for several minutes. The reaction mixture was left in the open vessel for one day to self-evaporate most of the Et_2_O (longer time in the open vessel did not result in the evaporation of more solvent). On the next day, the glassy body obtained was subjected to evaporation in a vacuum to remove remnant volatiles resulting in 0.310 g of the solid, yield: 98%.

^1^H NMR (300 MHz, CDCl3) δ 7.74–7.64 (m, 1H, Ar), 7.54–7.44 (m, 1H, Ar), 7.45–7.33 (m, 2H, Ar), 5.82 (s, 1H, CH), 3.18–3.05 (m, 4H, CH_2_), 3.03–2.83 (m, 4H, CH_2_). ^11^B NMR (96 MHz, CDCl_3_): δ (ppm) = 31. EA calculated for C_11_H_14_BNO_2_S: C(56.19%), H(6.00%), N(5.96%); determined: C(54.31%), H(6.55%), N(8.12%). m.p. = 56–57 °C (decomposition).

3-Piperidin-1-yl-2,1-benzoxaborol-1(3H)-ol (**9**)

(2-formylphenyl)boronic acid (0.255 g, 1.70 mmol) and diethyl ether (10 mL) were placed in a glass vial and mixed until dissolved. Piperidine (0.169 mL, 1.70 mmol) was added to the solution and mixed on a magnetic stirred for several minutes. The reaction mixture was left in the open vessel for one day to self-evaporate most of the Et_2_O (longer time in the open vessel did not result in the evaporation of more solvent). On the next day, the glassy body obtained was subjected to evaporation in a vacuum to remove remnant volatiles resulting in 0.353 g of the solid, yield: 95%.

^1^H NMR (300 MHz, CDCl_3_) δ 7.78–7.61 (m, br, 1H, Ar), 7.58–7.34 (m, 3H, Ar), 5.88 (s, br, 1H, CH), 2.72–2.52 (m, br, 4H), 1.67–1.48 (m, br, 6H). ^11^B NMR (96 MHz, CDCl_3_): δ (ppm) = 30. EA calculated for C_12_H_16_BNO_2_: C(66.40%), H(7.43%), N(6.45%); determined: C(67.30%), H(7.93%), N(7.51%). m.p. = 66–70 °C (decomposition).

3-(4-Methylpiperazin-1-yl)-2,1-benzoxaborol-1(3H)-ol (**10**)

A solution of (2-formylphenyl)boronic acid (1.5 g; 10 mmol) in diethyl ether (70 mL) was stirred in a 100 mL flask at room temperature. Next, 1-methylpiperazine (1.0 g; 1.11 mL; 10 mmol) was added during 4 min resulting immediately in precipitation of a white solid. It was filtered, washed with diethyl ether (2 × 3 mL) and dried to give the desired product. Due the presence of residual substrate in the product at a ratio of 0.18: 1, the contaminated product was crystallized in a mixture of CH_2_Cl_2_ (30 mL) and hexane (30 mL). It was again filtered, washed with diethyl ether (2 × 3 mL) and dried to give the title compound. Yield: 1.85 g; 8 mmol; 79%.

^1^H NMR (300 MHz, CDCl_3_) δ 7.71 (dt, *J* = 7.1, 1.0 Hz, 1H, Ar), 7.48–7.29 (m, 3H, Ar), 5.84 (s, 1H, CH), 3.04–2.39 (m, br, 8H, CH_2_), 2.33 (s, 3H, CH_3_). ^13^C NMR (75 MHz, CDCl_3_): *δ* 152.8; 130.7; 130.3; 128.2; 122.7; 96.5; 55.3; 45.9. ^11^B NMR (96 MHz, chloroform-d_6_): *δ* 31. EA calculated for C_12_H_17_N_2_B0_2_: C(62.10%), H(7.38%), N(12.07%); determined: C(62.08%), H(7.26%), N(12.13%). m.p. = 150–151 °C (decomposition).

3-(4-Phenylpiperazin-1-yl)-2,1-benzoxaborol-1(3H)-ol (**11**)

A solution of (2-formylphenyl)boronic acid (1.5 g; 10 mmol) in diethyl ether (70 mL) was stirred in a 100 mL flask at room temperature. Next, 1-phenylpiperazine (1.6 g; 1.5 mL; 10 mmol) was added during 5 min resulting immediately in precipitation of a white solid. It was filtered, washed with diethyl ether (2 × 3 mL) and dried to give the desired product. The crude product was crystallized in a mixture of CH_2_Cl_2_ (100 mL) and hexane (100 mL). It was again filtered, washed with diethyl ether (2 × 3 mL) and dried to give the title compound. Yield: 2.21 g, 7.5 mmol, 75%.

^1^H NMR (300 MHz, CDCl_3_) δ 7.72 (d, *J* = 7.0 Hz, 1H, Ar), 7.57–7.37 (m, 3H, Ar), 7.31–7.19 (m, 2H), 6.95–6.89 (m, 2H), 6.85 (t, *J* = 7.3 Hz, 1H), 5.98 (s; 1H), 3.18 (t, *J* = 5.0 Hz, 4H, CH_2_), 2.90–2.27 (m, 4H, CH_2_). ^13^C NMR (75 MHz, CDCl_3_): δ 152.6, 151.5, 131.4, 130.3, 129.2, 128.6, 123.0, 119.9, 116.4, 97.6, 49.6, 46.9. ^11^B NMR (96 MHz, CDCl_3_): *δ* 31. EA calculated for C_17_H_19_N_2_BO_2_: C(69.41%), H(6.51%), N(9.52%); determined: C(69.40%), H(6.48%), N(9.51%). m.p. = 149–151 °C (decomposition).

1-Hydroxy-3-(4-phenylpiperazin-1-yl)-1,3-dihydro-2,1-benzoxaborole-7-carbaldehyde (**14**)

A solution of (2,6-diformylphenyl)boronic acid (1.0 g; 5 mmol) in diethyl ether (180 mL) was stirred in a 250 mL flask at room temperature. Next, 1-phenylpiperazine (0.83 g; 0.78 mL; 5 mmol) was added during 3 min resulting in precipitation of a white solid after 2 h. It was filtered, washed with diethyl ether (2 × 3 mL) and dried to give the title compound. Yield: 0.80 g; 2 mmol; 49%.

^1^H NMR (300 MHz, CDCl_3_) δ 10.05 (s, 1H), 8.07 (s, 1H), 8.00–7.86 (m, 1H, Ar), 7.82–7.67 (m, 2H, Ar), 7.35–7.15 (m, 2H, Ar), 6.93–6.88 (m, 2H, Ar), 6.88–6.81 (m, 1H, Ar), 6.02 (s, 1H, CH), 3.17 (t, *J* = 5.1 Hz, 4H, CH_2_), 2.95–2.83 (m, 2H, CH_2_), 2.80–2.70 (m, 2H, CH_2_). ^13^C NMR (75 MHz, CDCl_3_): δ 196.3; 154.7; 151.4; 138.3; 135.2; 132.0; 129.4; 129.2; 119.8; 116.3; 97.6; 49.4; 47.0. ^11^B NMR (96 MHz, CDCl_3_): δ 31. EAsw calculated for C_18_H_19_N_2_B0_3_: C(67.11%) H(5.94%) B(3.36%) N(8.70%); determined: C(67.03%) H(5.97%) B(3.36%) N(8.79%). m.p. = 171–173 °C (decomposition).

1-Hydroxy-3-(4-methylpiperazin-1-yl)-1,3-dihydro-2,1-benzoxaborole-7-carbaldehyde (**15**)

A solution of (2,6-diformylphenyl)boronic acid (1.0 g; 5 mmol) in diethyl ether (180 mL) was stirred in a 250 mL flask at room temperature. Next, 1-methylpiperazine (0.5 g; 0.56 mL; 5 mmol) was added during 2 min resulting in precipitation of a white solid after 2 h. It was filtered, washed with diethyl ether (2 × 3 mL) and dried to give the title compound. Yield: 1.1 g; 4.2 mmol; 83%.

^1^H NMR (300 MHz, CDCl_3_) δ 10.11 (s, 1H, CHO), 8.01–7.83 (m, 1H, Ar), 7.75–7.66 (m, 2H, Ar), 5.95 (s, 1H, CH), 2.89–2.33 (m, br, 8H, CH_2_), 2.28 (s, 3H, CH_3_). ^13^C NMR (126 MHz, CDCl_3_): *δ* 195.8; 154.6; 138.3; 133.9; 131.7; 129.1; 97.3; 55.2; 46.0. ^11^B NMR (96 MHz, CDCl_3_): *δ* 31. EA calculated for C_13_H_17_N_2_BO_3_: C(60.03%), H(6.59%), N(10.77%); determined: C(60.02%), H(6.63%), N(10.70%). m.p. = 173–176 °C (decomposition, browning).

## Figures and Tables

**Table 1:** The average diameter of the zone of totally inhibited growth of *Candida albicans* at various amounts (mm) and Minimal Inhibitory Concentration (μg/mL).

Compound	10 μg	25 μg	50 μg	100 μg	DMSO	MIC (μg/mL)
amphotericine B	9 ± 1	9 ± 1	8 ± 1	9 ± 1	0	<=1
Tavaborole [31]	40 ± 1	44 ± 1	48 ± 2	54 ± 2	0	2
**1**	20 ± 1	25 ± 2	28 ± 2	31 ± 3	0	62.5
**2**	20 ± 1	25 ± 1	27 ± 2	29 ± 1	0	62.5
**3**	20 ± 2	25 ± 1	27 ± 2	31 ± 1	0	62.5
**4**	12 ± 1	27 ± 1	30 ± 1	34 ± 1	0	31.2
**5**	19 ± 1	25 ± 2	28 ± 1	32 ± 1	0	31.2
**6**	30 ± 1	36 ± 1	39 ± 1	44 ± 2	0	8
**7**	11 ± 1	17 ± 1	23 ± 1	27 ± 1	0	125
**8**	7 ± 1	13 ± 2	20 ± 2	25 ± 2	0	125
**9**	10 ± 1	16 ± 2	22 ± 1	26 ± 2	0	125
**10**	6 * ± 1	14 ± 1	21 ± 1	28 ± 1	0	62.5
**11**	5 * ± 1	10 ± 1	18 ± 1	21 ± 1	0	62.5
**12**	5 * ± 1	15 ± 1	22 ± 2	26 ± 1	0	62.5
**13**	12 ± 1	18 ± 2	22 ± 1	27 ±1	0	62.5
**14**	0	0	0	0	0	>250
**15**	0	0	0	0	0	>500

* only inhibition of the growth of *C. albicans* was observed in that case.

## Supplementary Materials

The following are available online. Structures of investigated compounds (Figures S1 and S2). Crystal data and structure refinement for **11** and **14** (Table S1). Selected geometrical parameters for **11** and **14** (Table S2). NMR spectra of **1**–**11**, **14** and **15**.
