# Supplementary material for: Synthesis and Influence of 3-Amino Benzoxaboroles Structure on Their Activity against Candida albicans
Source: Molecules. 2020 Dec 18;25(24):5999. doi: 10.3390/molecules25245999 (PMC7766895; doi:10.3390/molecules25245999)
Supplement: Supplementary file 1 [file molecules-25-05999-s001.pdf]

Supplementary Materials

of the manuscript entitled:

Synthesis and Influence of 3-Amino Benzoxaboroles Structure on Their Activity against *Candida albicans*

Dorota Wieczorek,[1] Ewa Kaczorowska,[2] Marta Wiśniewska,[2] Izabela D. Madura,[2] Magdalena Leśniak,[2] Jacek Lipok,[1] Agnieszka Adamczyk-Woźniak[2]*

[1] Faculty of Chemistry, University of Opole, Oleska 48, 45-052 Opole, Poland,
[2] Faculty of Chemistry, Warsaw University of Technology, Noakowskiego 3, 00-664 Warsaw, Poland
*Corresponding author: agnieszka@ch.pw.edu.pl

Table of contents:

1. Compounds studied (Figs. S1 and S2).
2. Crystal data and structure refinement for **11** and **14** (Table S1).
3. Selected geometrical parameters for **11** and **14** (Table S2).
4. NMR spectra of compounds studied (Figs. S3- SNr)

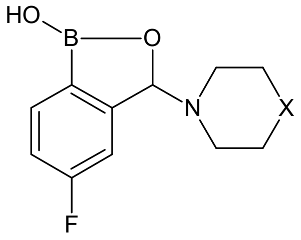

1 (X = O), **2** (X = S), **3** (X = CH_2)

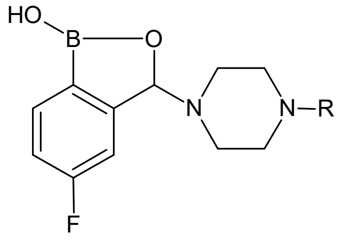

4 (R = Me), **5** (R = Ph)

Fig. S1. *Structures of studied Tavaborole's derivatives (1-5).*

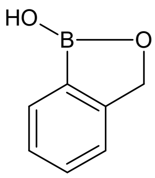

6

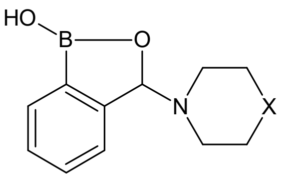

7 (X = O), **8** (X = S), **9** (X = CH_2)

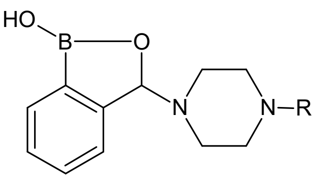

10 (R = Me), **11** (R = Ph),
12 (R = 2-F-Ph), **13** (R = 4-F-Ph)

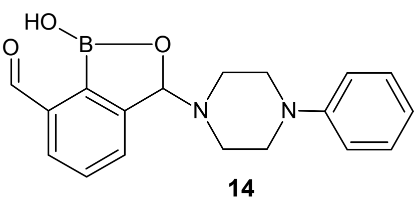

14

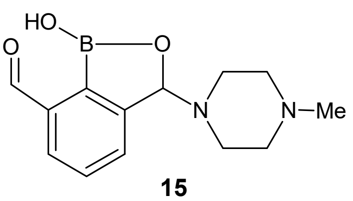

15

Fig. S2. *Structures of unsubstituted benzoxaborole (**6**) and its studied derivatives (**7-15**).*

Table **S1**. Crystal data and structure refinement for **11** and **14**.

	**11**	**14**
CCDC numer	1998838	1998837
Empirical formula	$C_{17}H_{19}BN_2O_2$	$C_{18}H_{19}BN_2O_3$
Formula weight	294.15	322.16
Temperature/K	295(2)	
Crystal system	Monoclinic	
Space group	$P2_1/n$	
a/Å	15.5396(5)	7.9960(4)
b/Å	6.1216(2)	5.8148(3)
c/Å	15.7458(5)	35.5074(16)
α/°	90	90
β/°	90.458(3)	96.257(4)
γ/°	90	90
Volume/Å^3	1497.81(8)	1641.09(14)
Z	4	4
ρ_{calc} g/cm^3	1.304	1.304
μ/mm^{-1}	0.085	0.088
F(000)	624.0	680.0
Radiation	Mo*Kα* (λ = 0.71073 Å)	
2Θ range for data collection/°	7.142 to 58.996	7.378 to 61.774
Index ranges	$-21 \leq h \leq 21$,	$-10 \leq h \leq 11$,
	$-8 \leq k \leq 8$,	$-8 \leq k \leq 7$,
	$-21 \leq l \leq 21$	$-40 \leq l \leq 47$
Reflections collected	40309	16368
Independent reflections	4187	4653
	[R_{int} = 0.0453,	[R_{int} = 0.0310,
	R_{sigma} = 0.0242]	R_{sigma} = 0.0319]
Data/restraints/parameters	4187/0/202	4653/0/220
Goodness-of-fit on F2	1.027	1.056
Final R indexes [I >2*σ* (I)]	R_1 = 0.0439, *wR2* = 0.0979	R_1 = 0.0573, *wR2* = 0.1234
Final R indexes [all data]	R_1 = 0.0623, *wR2* = 0.1087	R_1 = 0.0894, *wR2* = 0.1401
Largest diff. peak/hole / e Å^{-3}	0.30/–0.20	0.15/–0.15

Table **S2**. Selected geometrical parameters for **11** and **14**.

	**11**	**14**		**11**	**14**
		Bond lengths / Å			
C1–B1	1.5620(19)	1.564(2)	C1–C2	1.3904(17)	1.381(2)
O1–B1	1.3493(17)	1.341(2)	C1–C6	1.3960(17)	1.411(2)
O2–B1	1.3787(17)	1.373(2)	C2–C3	1.3862(17)	1.394(2)
O2–C7	1.4627(15)	1.4562(18)	C2–C7	1.5122(16)	1.505(2)
O3–C18		1.206(3)	C3–C4	1.3839(19)	1.378(3)
N1–C7	1.4386(14)	1.4310(19)	C4–C5	1.382(2)	1.376(3)
N1–C8	1.4635(15)	1.458(2)	C5–C6	1.380(2)	1.386(3)
N1–C11	1.4614(15)	1.4571(18)	C6–C18		1.458(3)
N2–C9	1.4730(15)	1.4618(18)	C8–C9	1.5174(16)	1.506(2)
N2–C10	1.4802(15)	1.4580(19)	C10–C11	1.5098(17)	1.504(2)
N2–C12	1.4370(14)	1.4028(18)			
		Bond angles / °			
B1–O2–C7	110.75(9)	111.11(12)	C2–C1–C6	118.39(12)	118.46(15)
O1–B1–O2	123.12(12)	118.40(15)	C2–C1–B1	104.69(10)	104.72(14)
O1–B1–C1	128.28(12)	133.50(16)	C6–C1–B1	136.83(12)	136.82(17)
O2–B1–C1	108.59(11)	108.08(14)	C1–C2–C7	111.31(10)	111.53(14)
C7–N1–C8	114.38(9)	114.13(11)	C3–C2–C1	122.23(12)	122.25(17)
C7–N1–C11	113.88(9)	114.13(11)	C3–C2–C7	126.43(12)	126.21(17)
C11–N1–C8	109.76(9)	108.54(12)	O2–C7–C2	104.62(9)	104.49(13)
C9–N2–C10	109.25(9)	112.70(12)	N1–C7–O2	112.96(9)	113.37(12)
C12–N2–C9	115.80(9)	117.64(11)	N1–C7–C2	112.90(9)	113.59(12)
C12–N2–C10	111.47(9)	117.20(11)			
		Torsion angles / °			
C8–N1–C7–O2	−77.8(1)	−69.5(2)	C2–C1–B1–O1	−179.4(1)	−179.0(2)
C8–N1–C7–C2	163.8(1)	171.4(1)	C2–C1–B1–O2	−0.6(1)	−0.9(2)
N1–C8–C9–N2	−61.3(1)	−56.3(2)	C6–C1–B1–O1	4.2(2)	0.6(3)
N2–C10–C11–N1	56.9(1)	56.1(2)	C6–C1–B1–O2	−177.0(1)	178.7(2)
C9–N2–C12–C13	−13.7(2)	19.5(2)	C7–O2–B1–O1	178.2(1)	−179.1(1)
C9–N2–C12–C17	166.1(1)	−162.5(2)	C7–O2–B1–C1	−0.7(1)	2.5(2)
C10–N2–C12–C13	112.0(1)	158.9(2)	B1–C1–C6–C18		1.7(3)
C10–N2–C12–C17	−68.23(14)	−23.2(2)	C1–C6–C18–O3		0.4(3)

NMR spectra of compound **1**

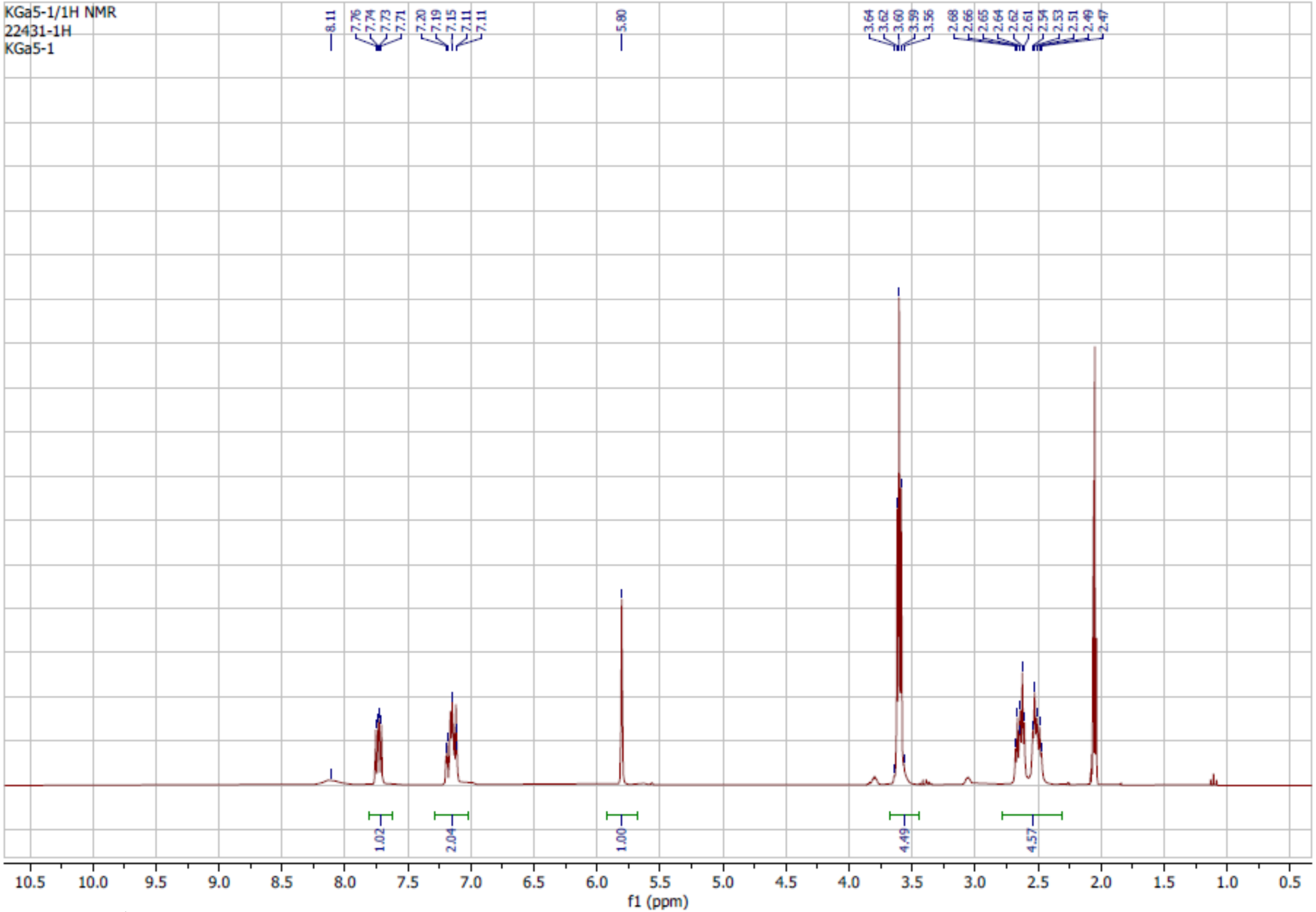

Fig. S.3. *^{1}H NMR spectrum of compound **1** in $(CD_3)_2CO$.*

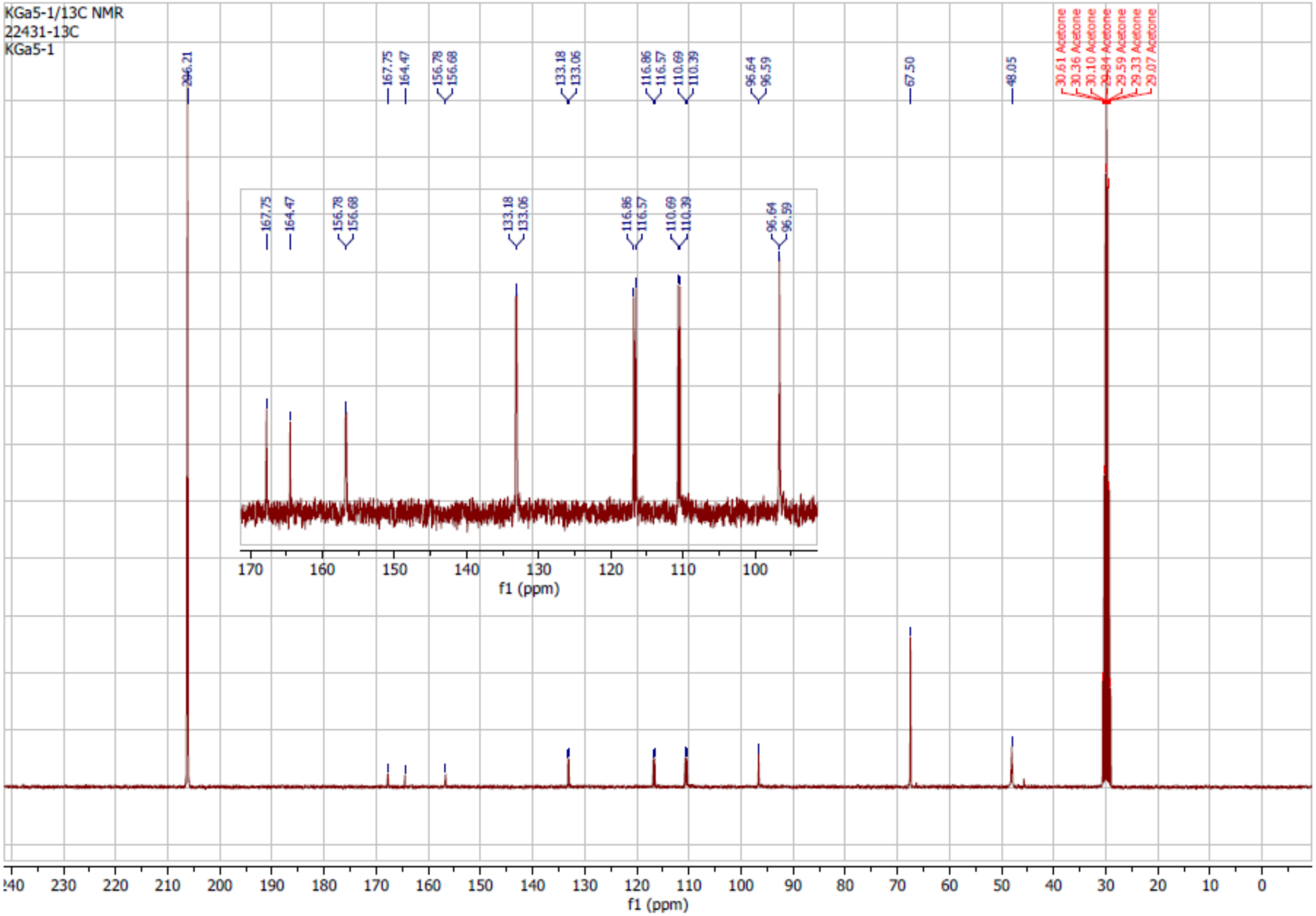

Fig. S.4. *^{13}C NMR spectrum of compound **1** in $(CD_3)_2CO$.*

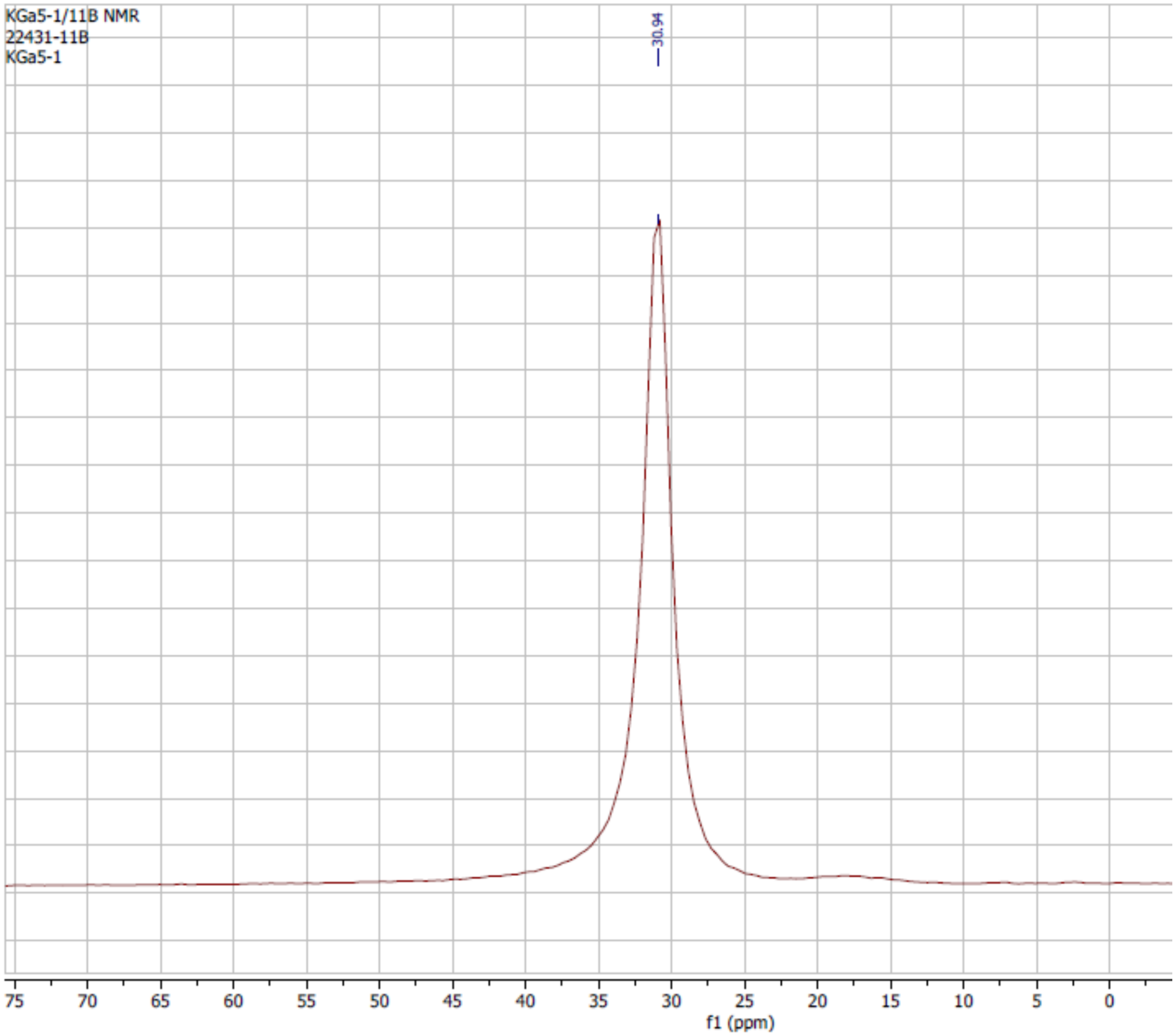

Fig. S.5. *^{11}B NMR spectrum of compound **1** in $(CD_3)_2CO$.*

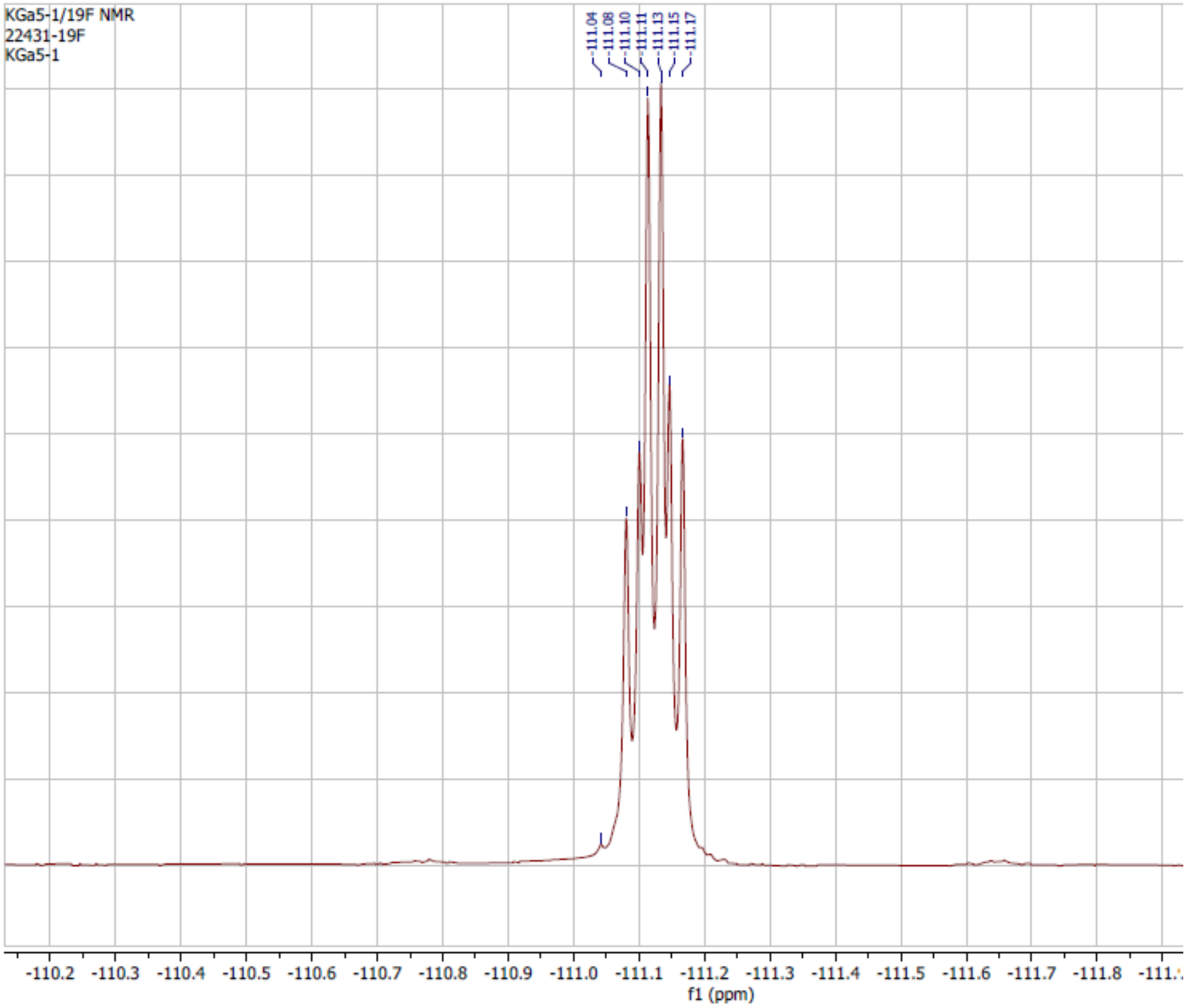

Fig. S.6. *^{19}F NMR spectrum of compound **1** in $(CD_3)_2CO$.*

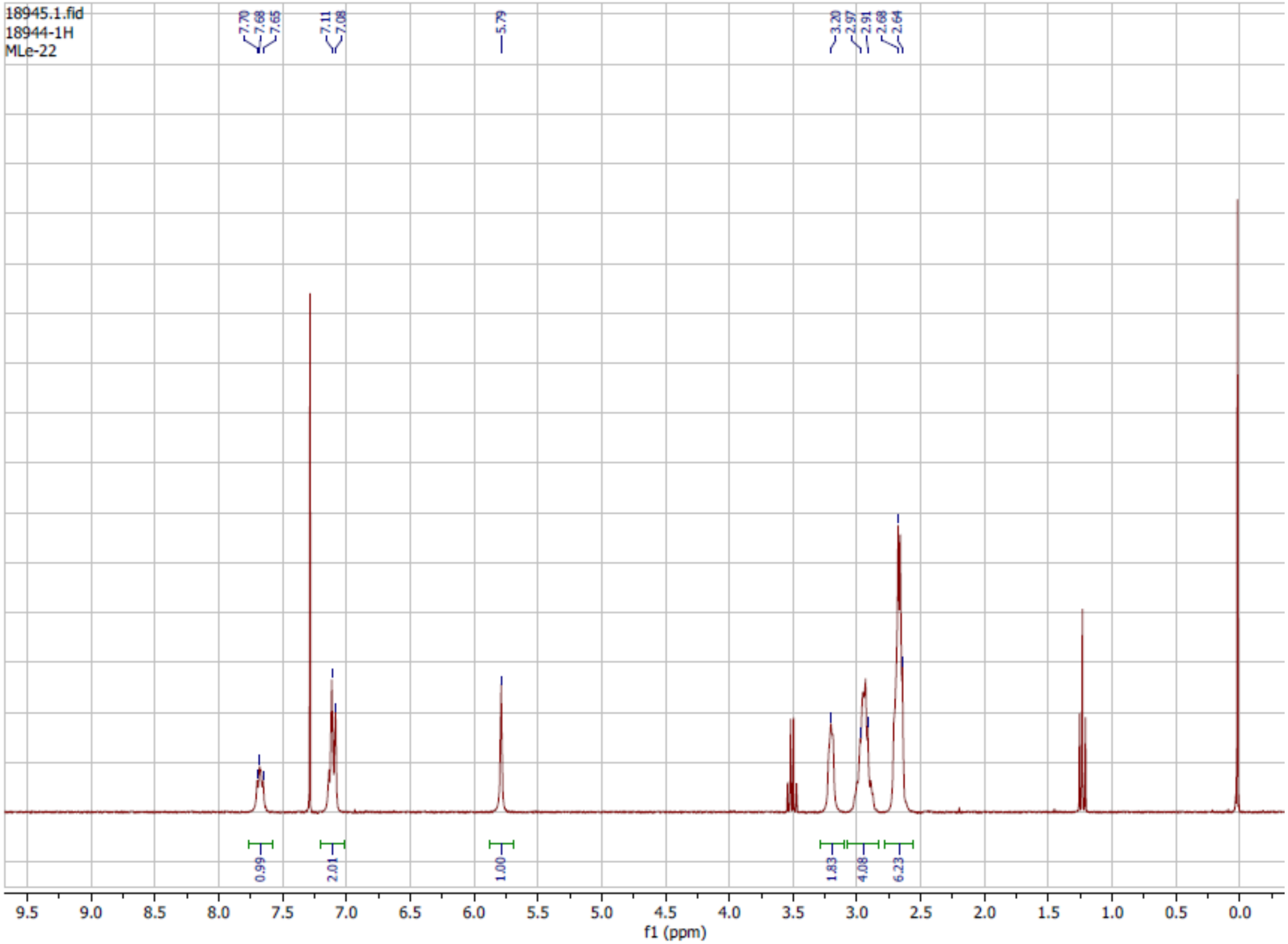

Fig. S.7. *^{1}H NMR spectrum of compound **2** in CDCl$_3$*

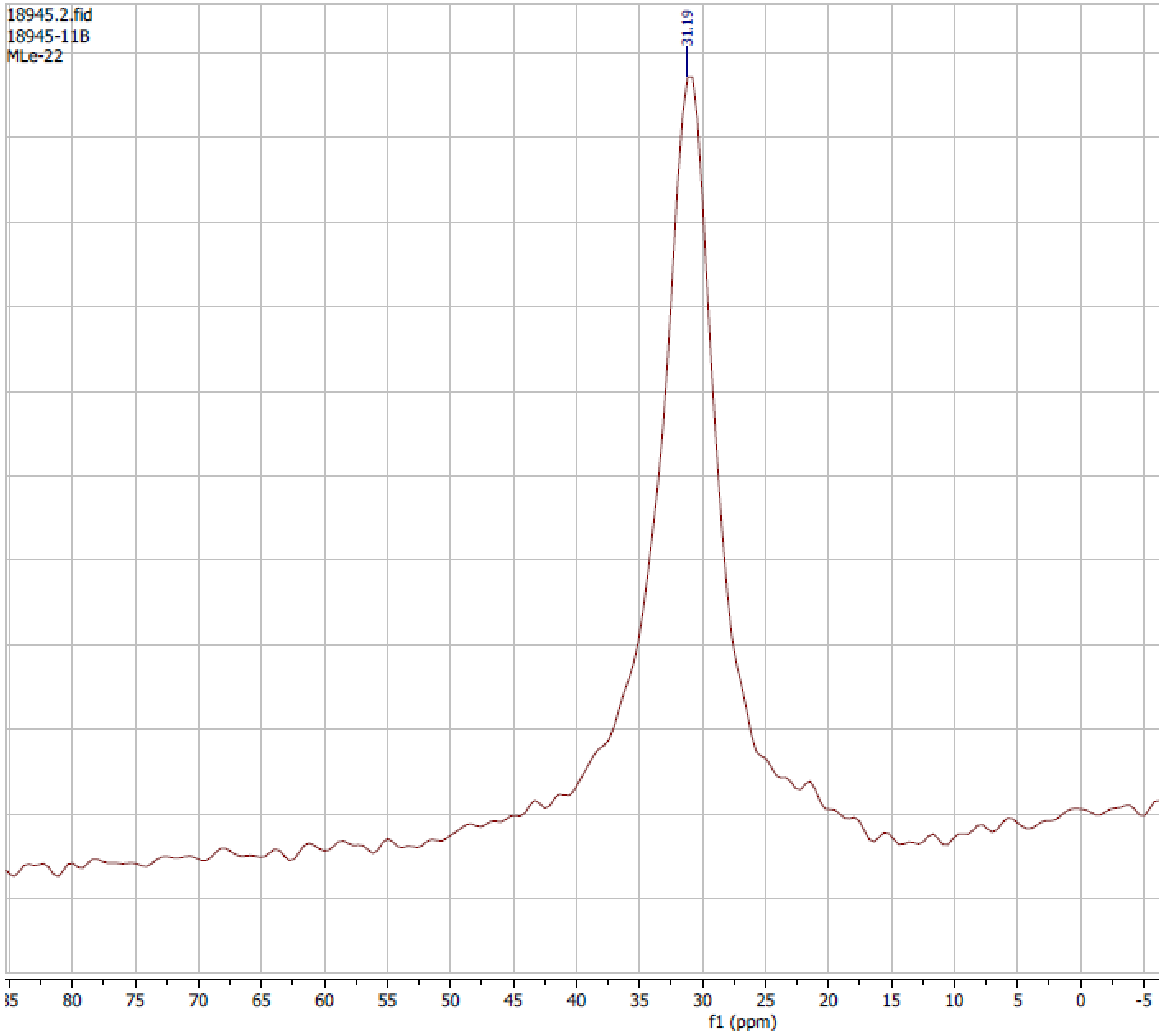

Fig. S.8. *^{11}B NMR spectrum of compound **2** in $CDCl_3$*

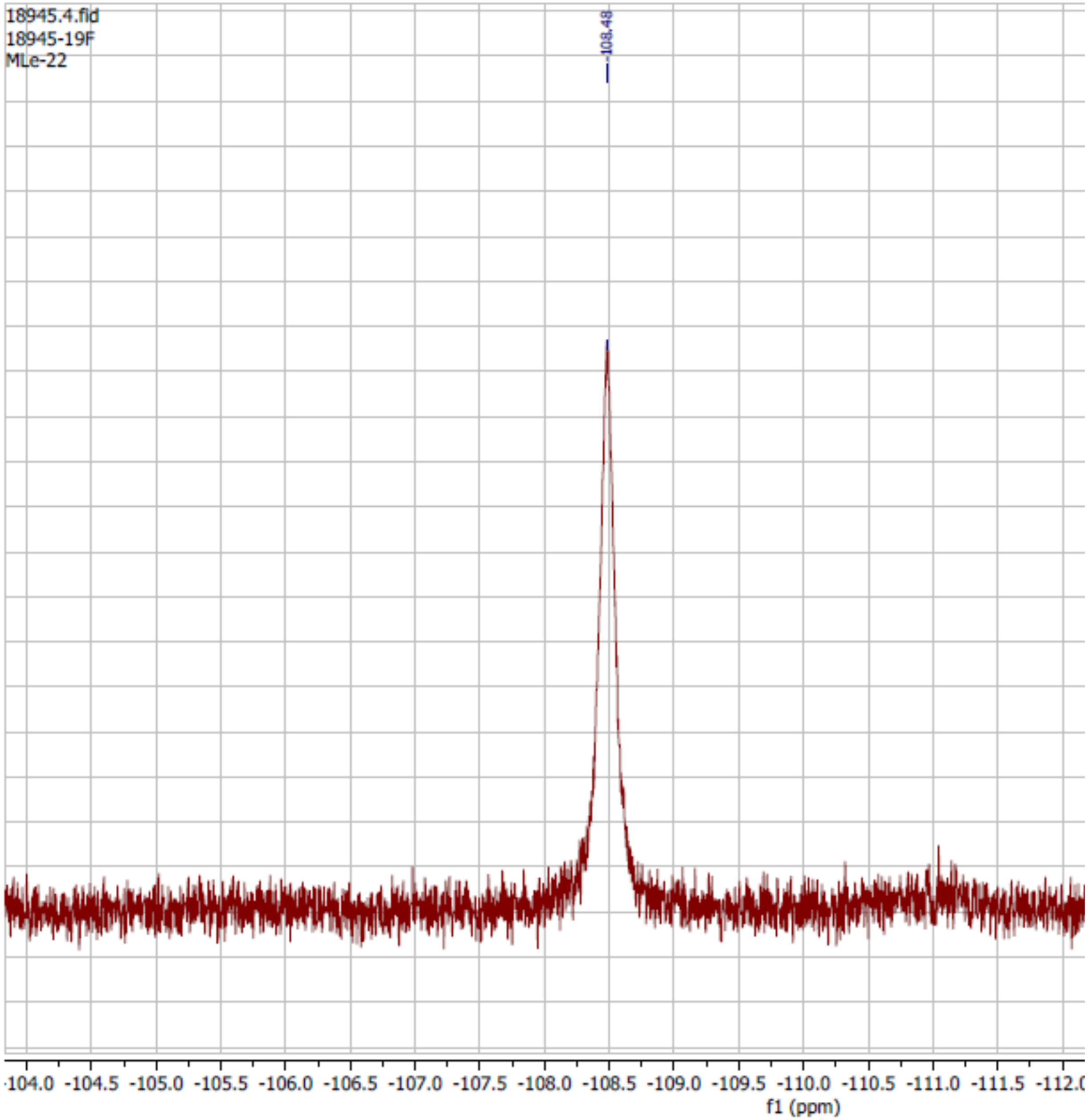

Fig. S.9. *^{19}F NMR spectrum of compound **2** in $CDCl_3$*

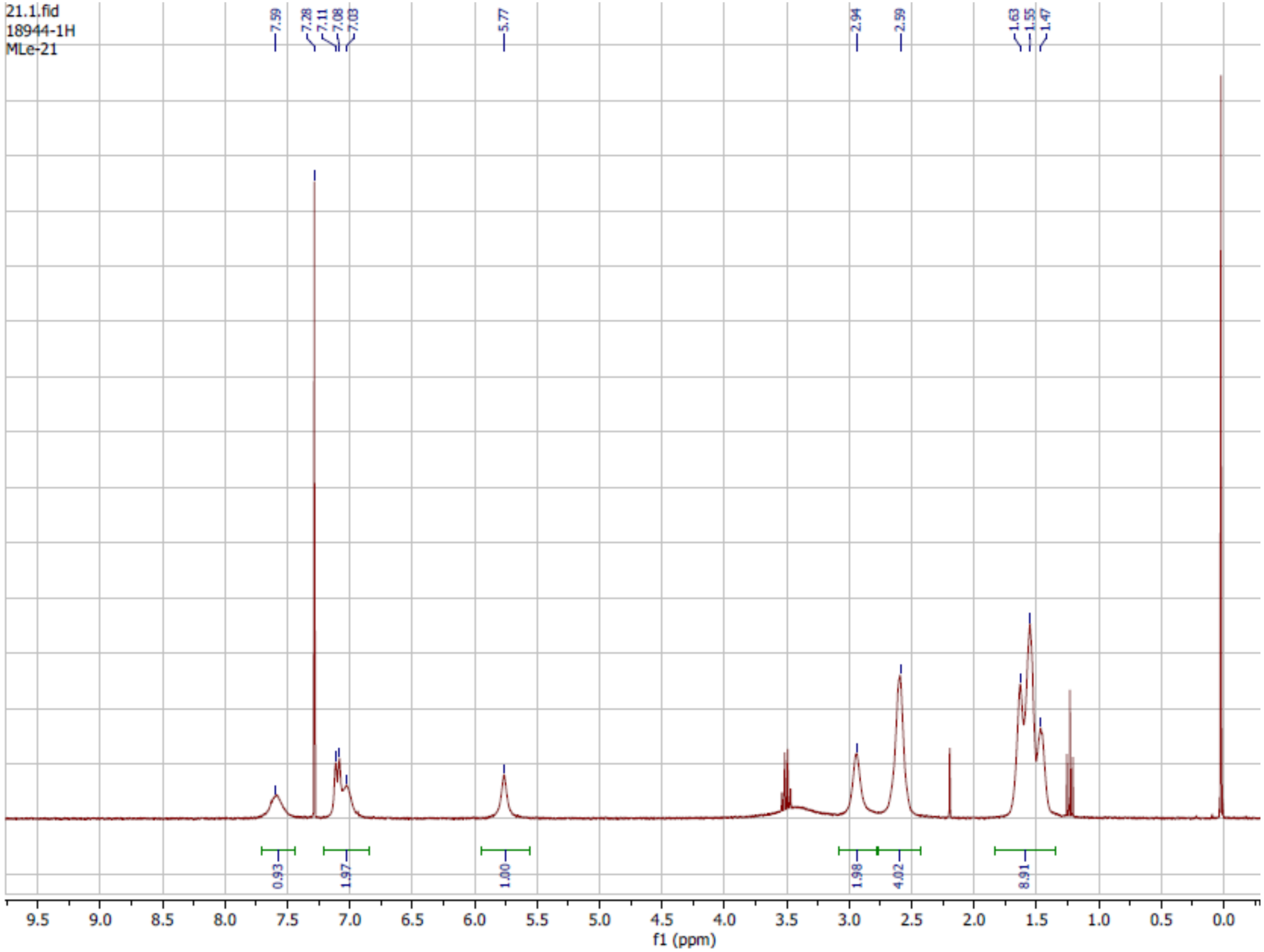

Fig. S.10. *^{1}H NMR spectrum of compound **3** in $CDCl_3$.*

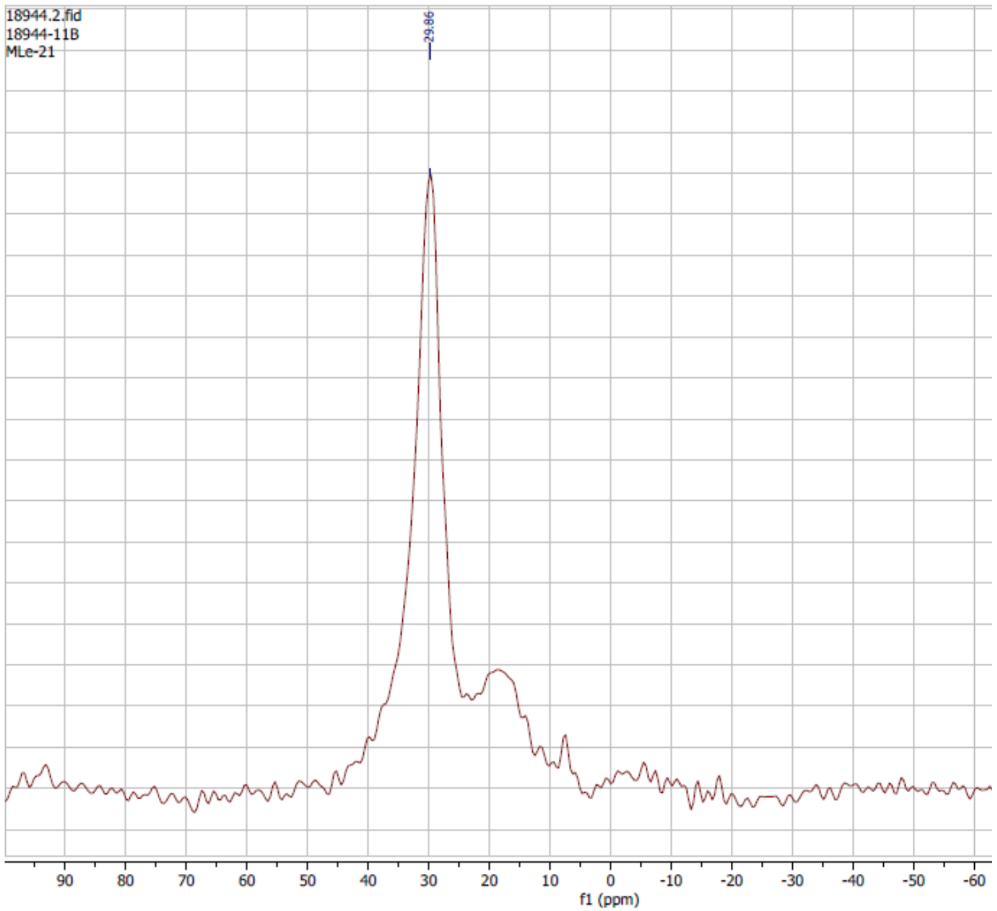

Fig. S.11. *^{11}B NMR spectrum of compound **3** in $CDCl_3$.*

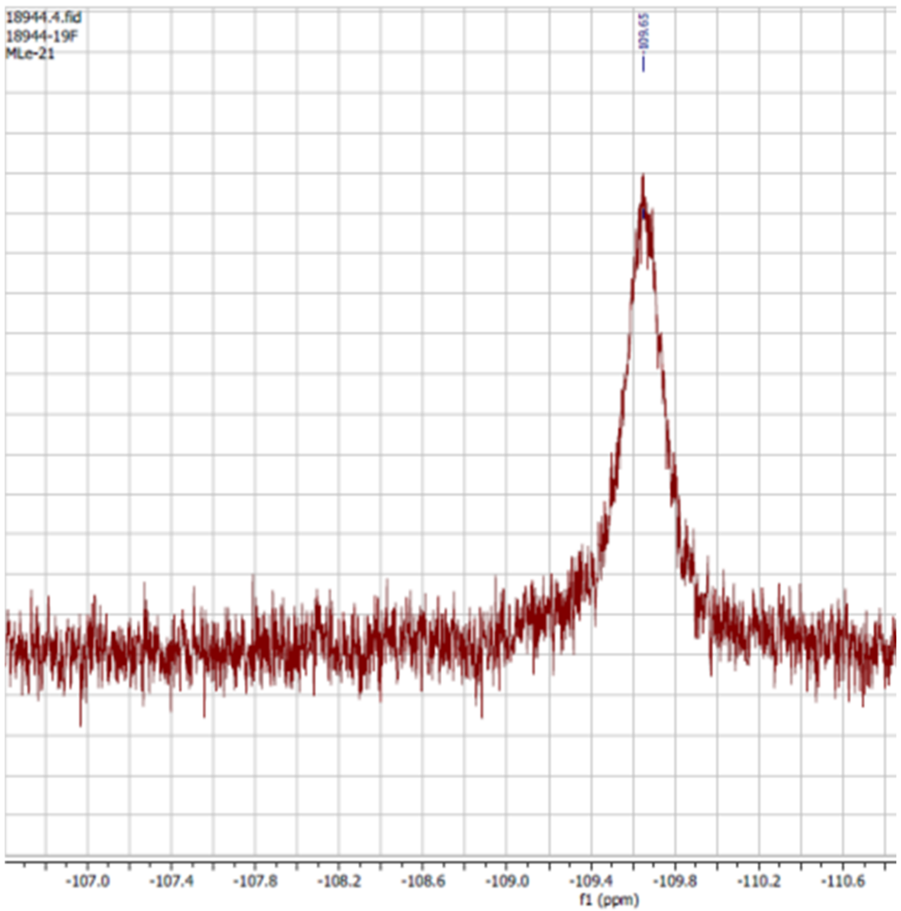

Fig. S.12*. ^{19}F NMR spectrum of compound **3** in $CDCl_3$*

NMR spectra of compound **4**

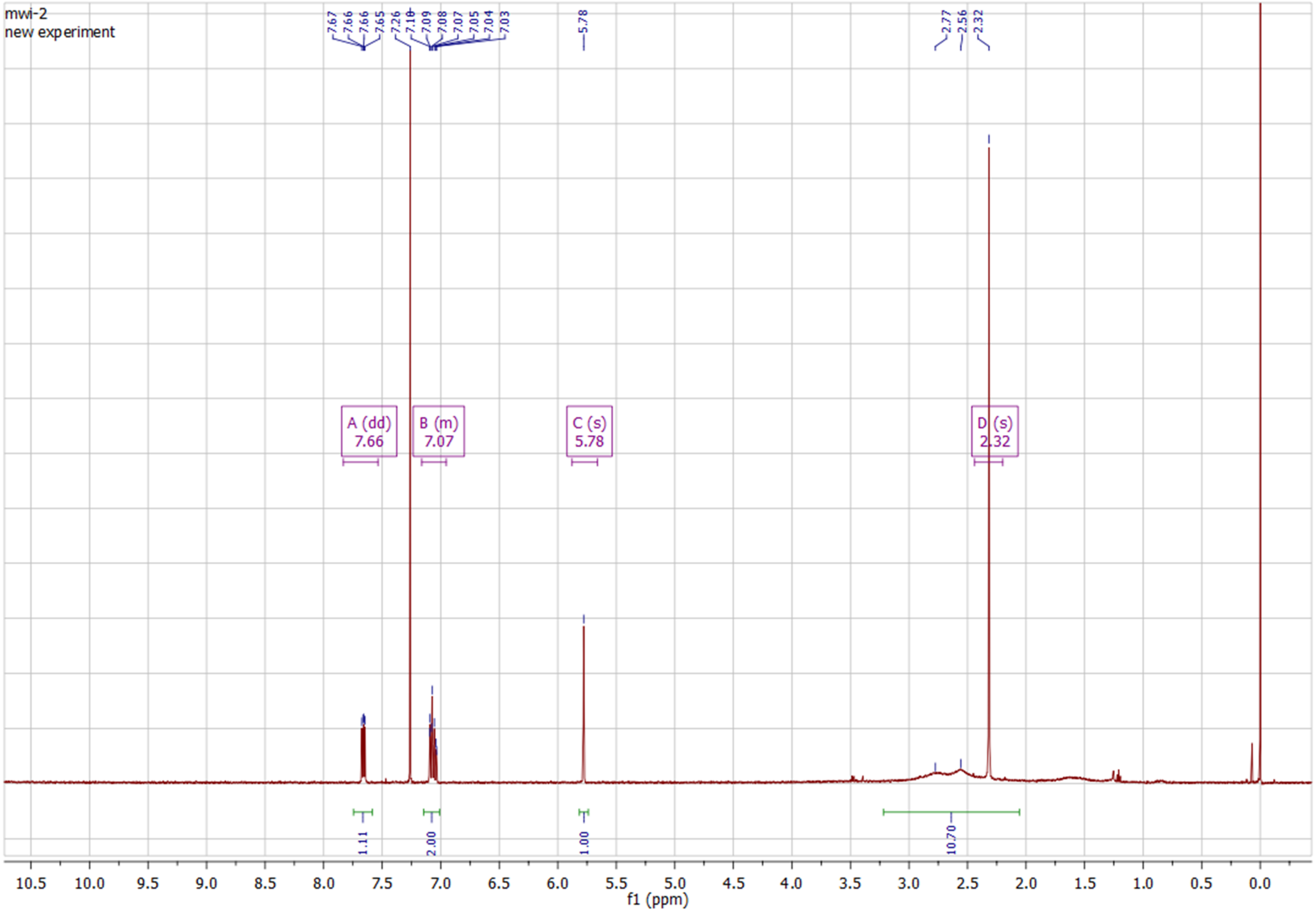

Fig. S.13. *^{1}H NMR spectrum of compound **4** in $CDCl_3$.*

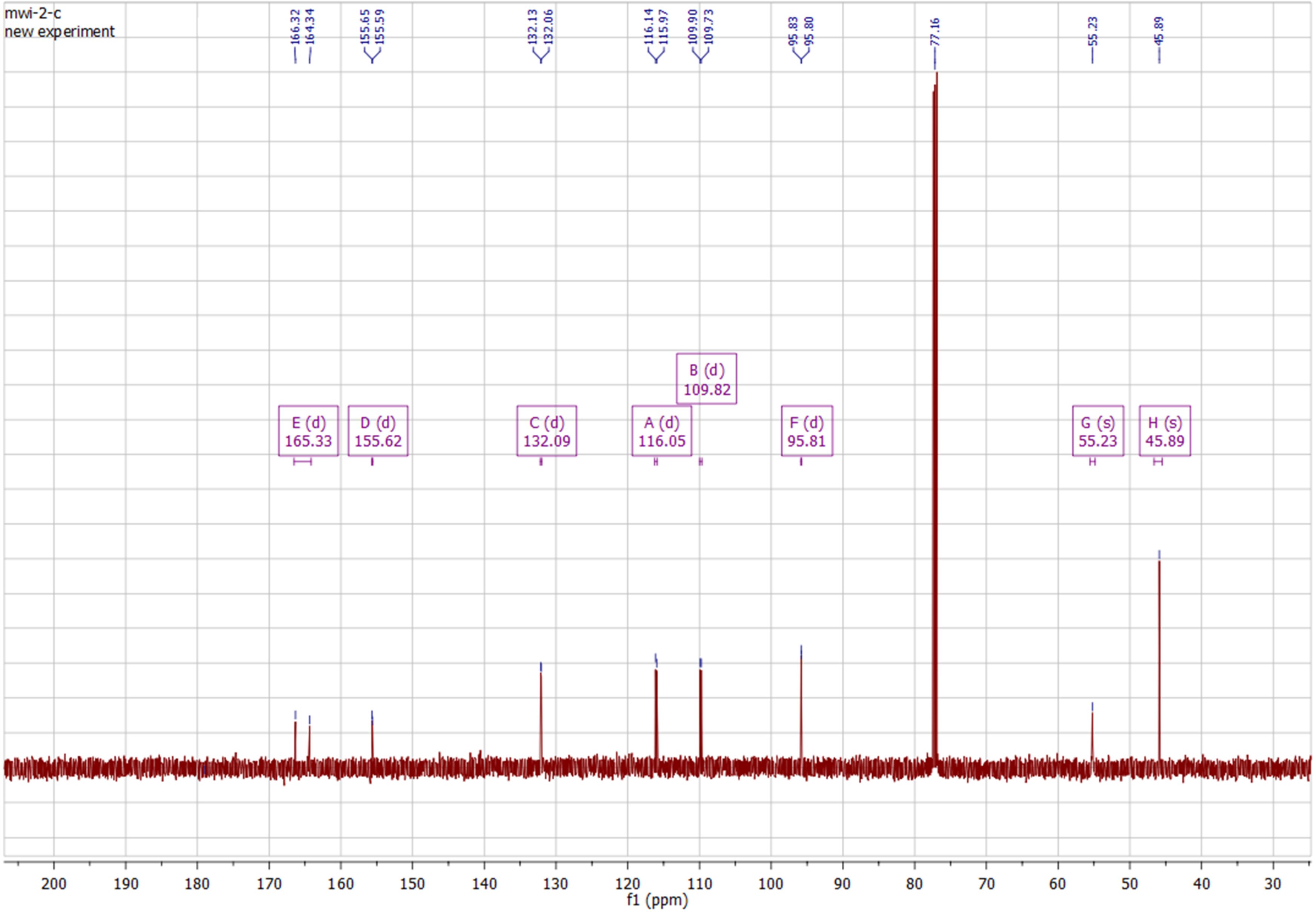

Fig. S.14. *[13] C NMR spectrum of compound **4** in $CDCl_3$.*

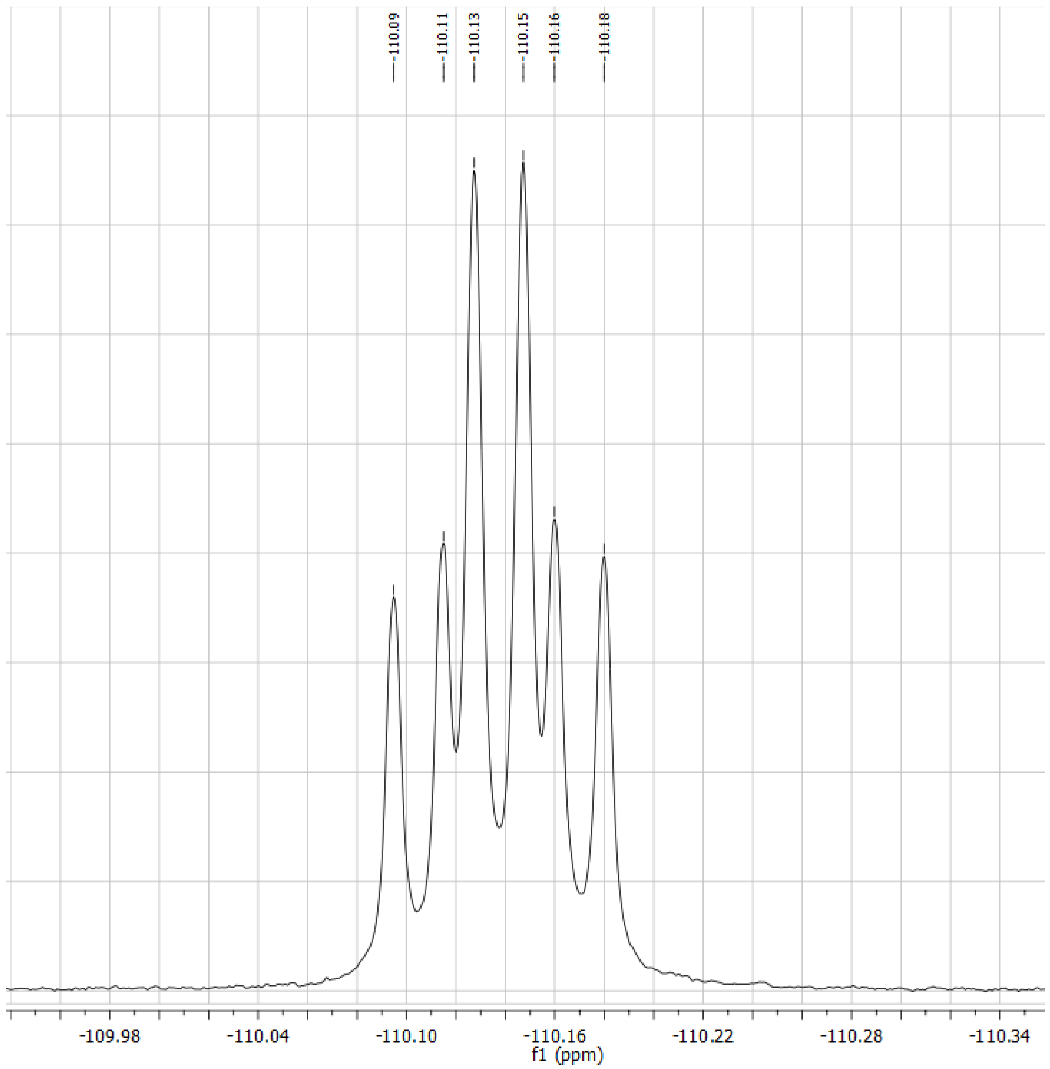

Fig. S.15. *^{19}F NMR spectrum of compound **4** in $CDCl_3$.*

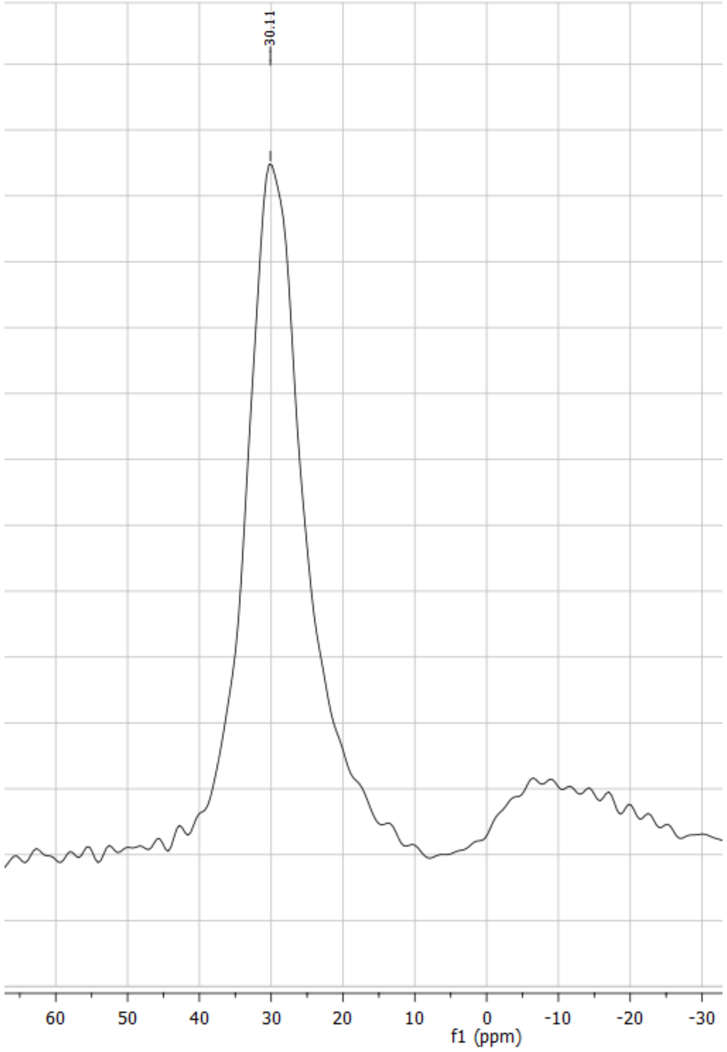

Fig. S.16. *^{11}B NMR spectrum of compound **4** in $CDCl_3$.*

NMR spectra of compound **5**

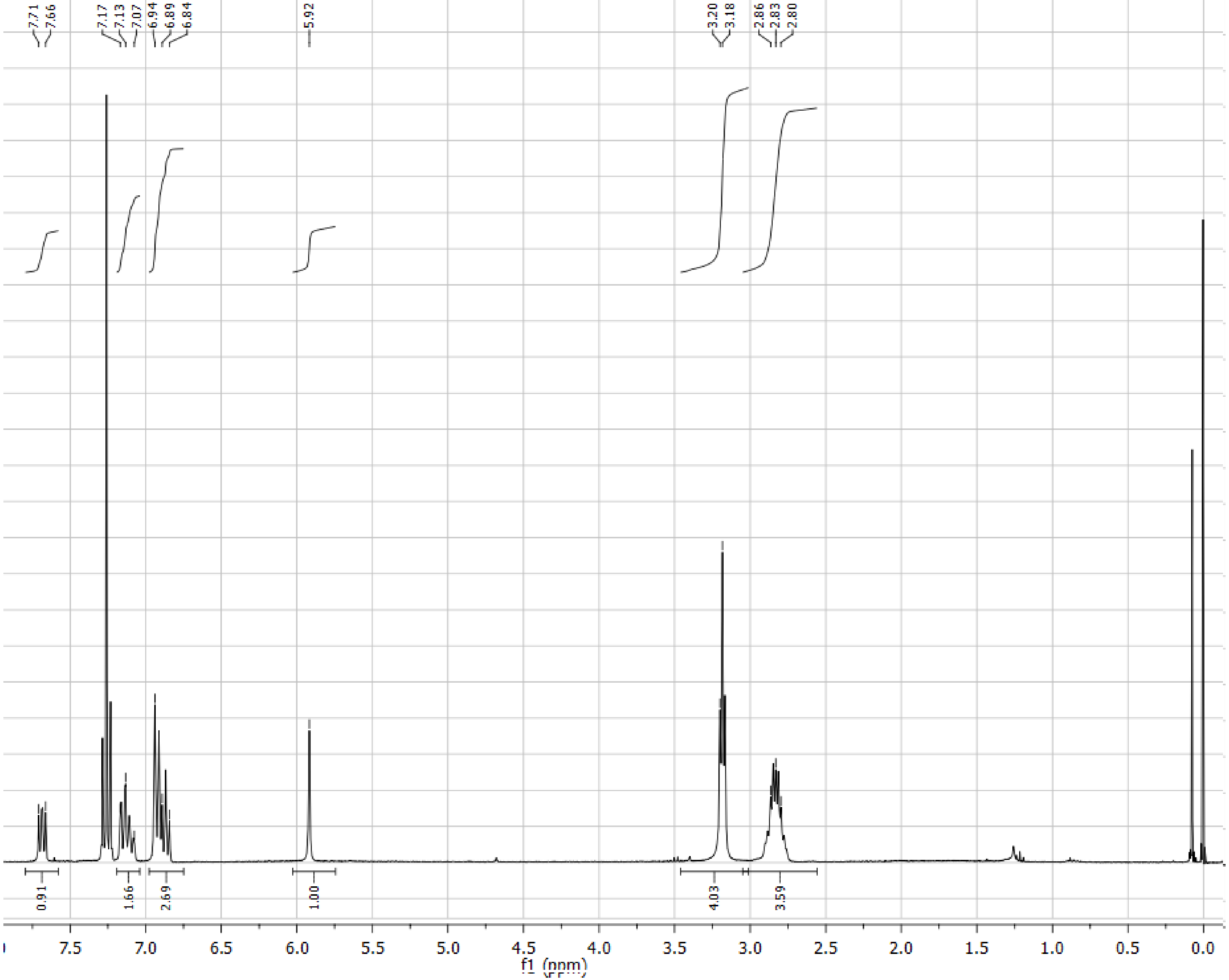

Fig. S.17. *^{1}H NMR spectrum of compound* ***5*** *in* $CDCl_3$.

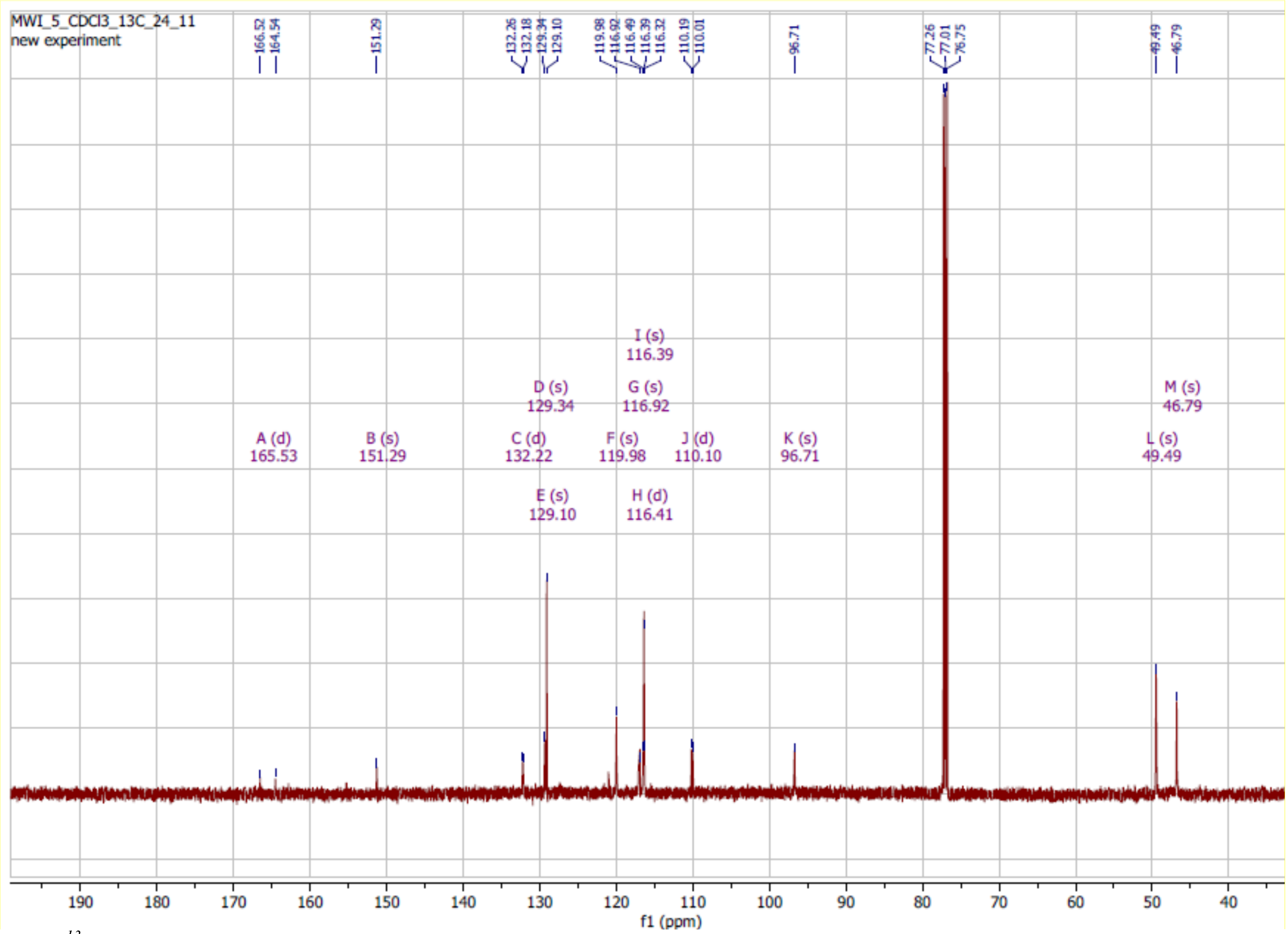

Fig. S.18. *^{13} CNMR spectrum of compound **5** in $CDCl_3$.*

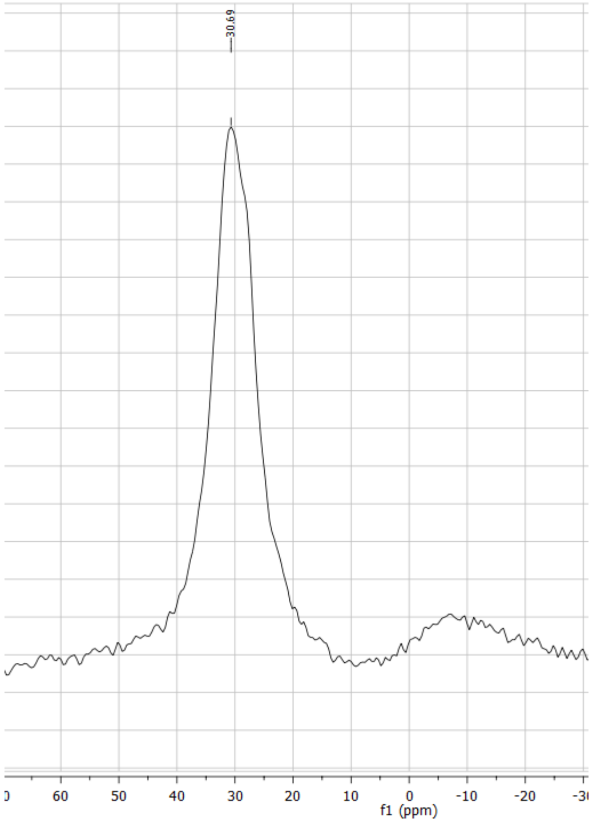

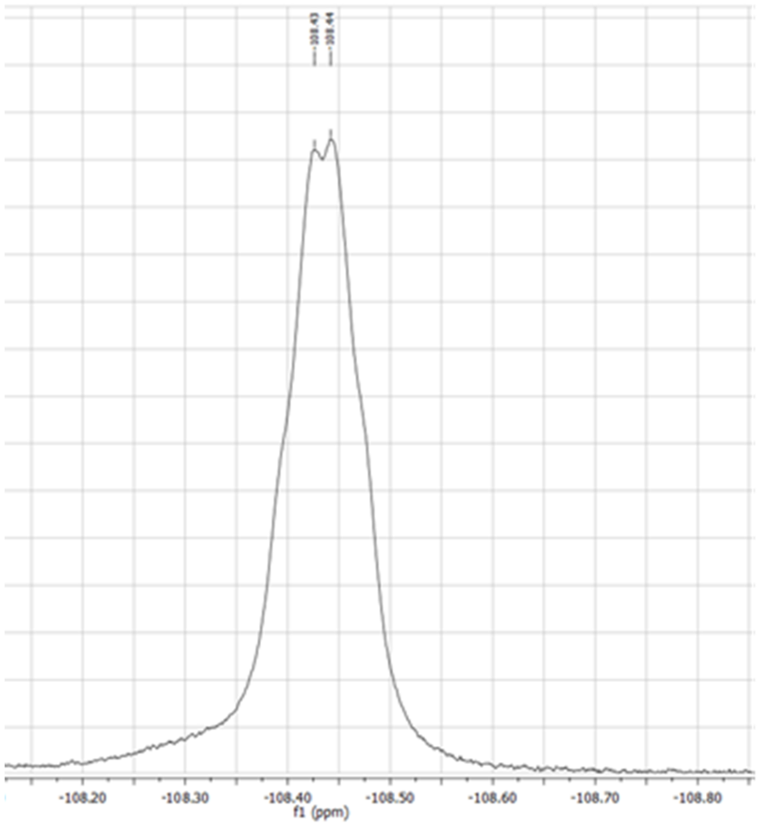

Fig. S.19. *^{11}B NMR spectrum of compound* ***5*** *in $CDCl_3$.*

Fig. S.20. *^{19}F NMR spectrum of compound* ***5*** *in $CDCl_3$.*

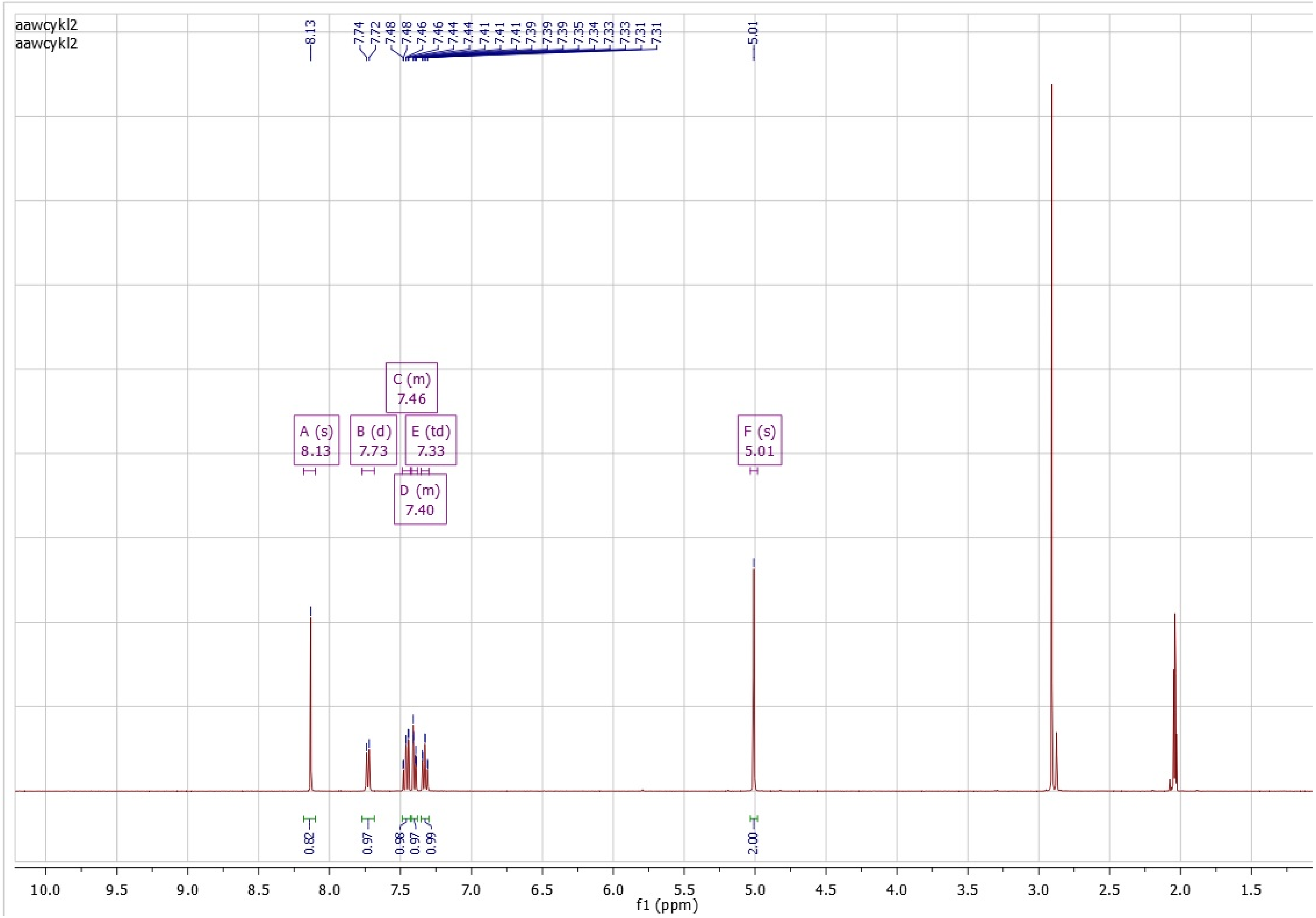

Fig. S.21. *^{1}H NMR spectrum of compound **6** in $(CD_3)_2CO$*

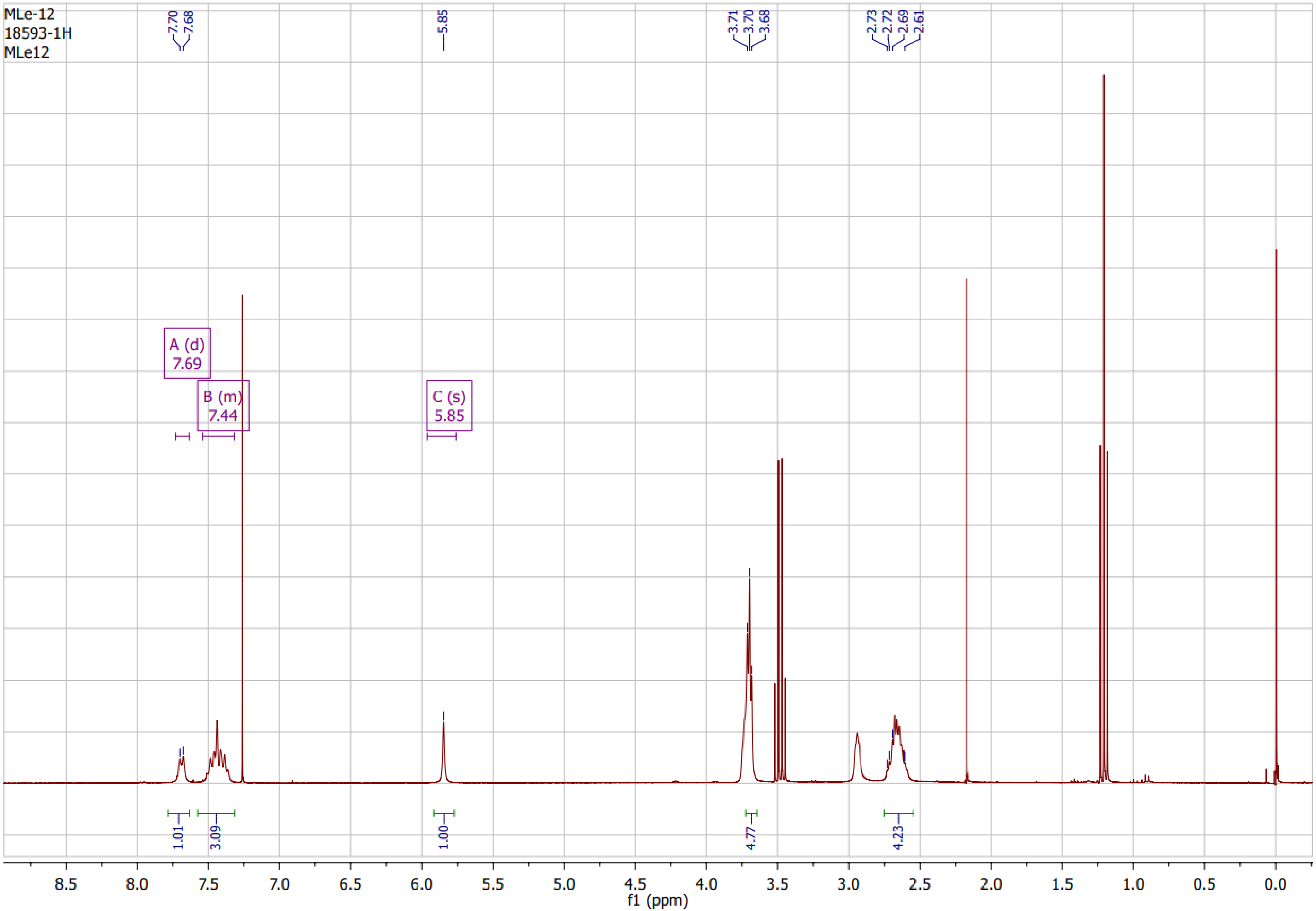

Fig. S.22. *[1] H NMR spectrum of compound 7 in $CDCl_3$.*

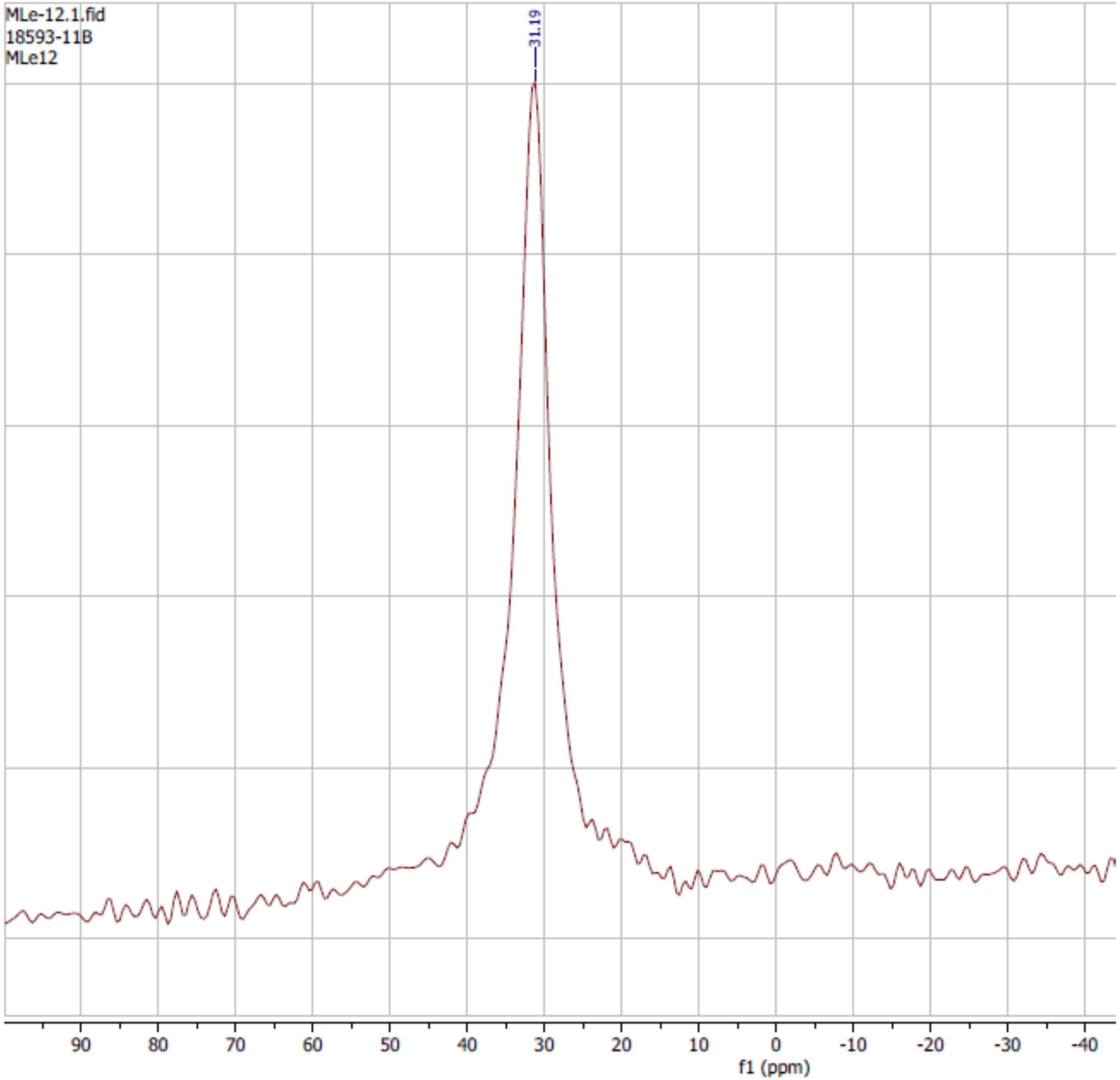

Fig. S.23. *[11] B NMR spectrum of compound **7** in $CDCl_3$*

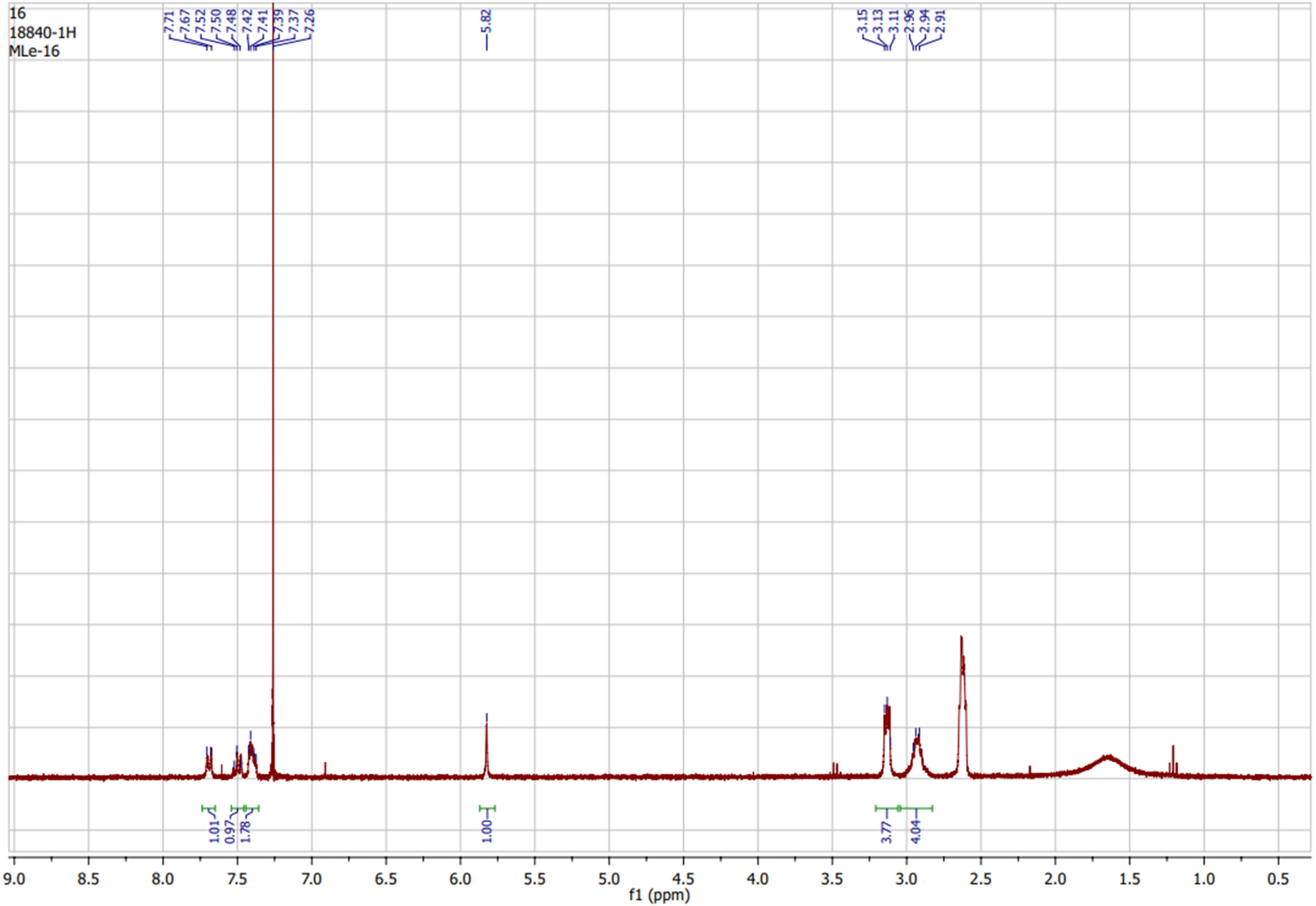

Fig. S.24. *[1] H NMR spectrum of compound **8** in CDCl₃*

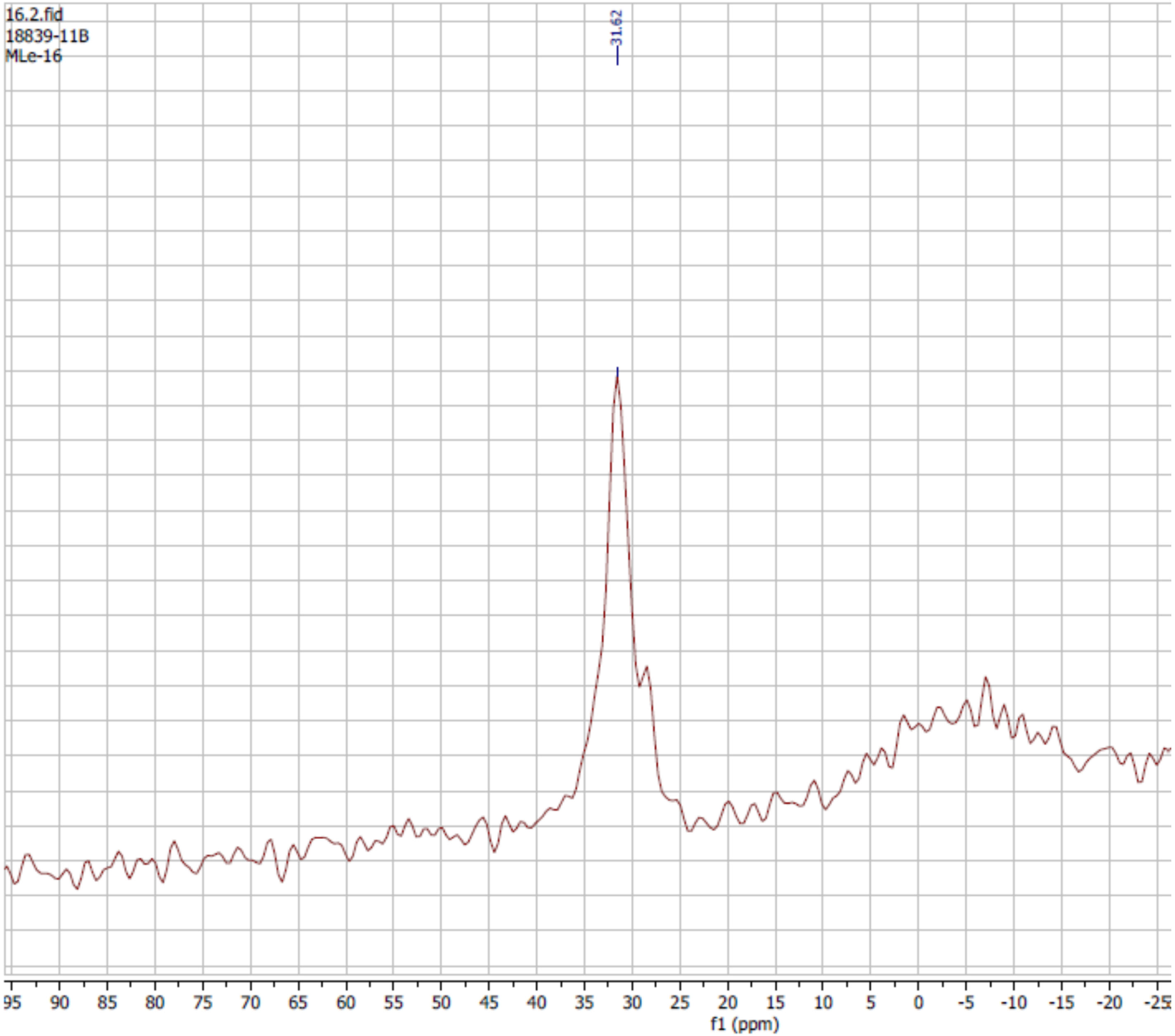

Fig. S.25. *[11] B NMR spectrum of compound **8** in $CDCl_3$*

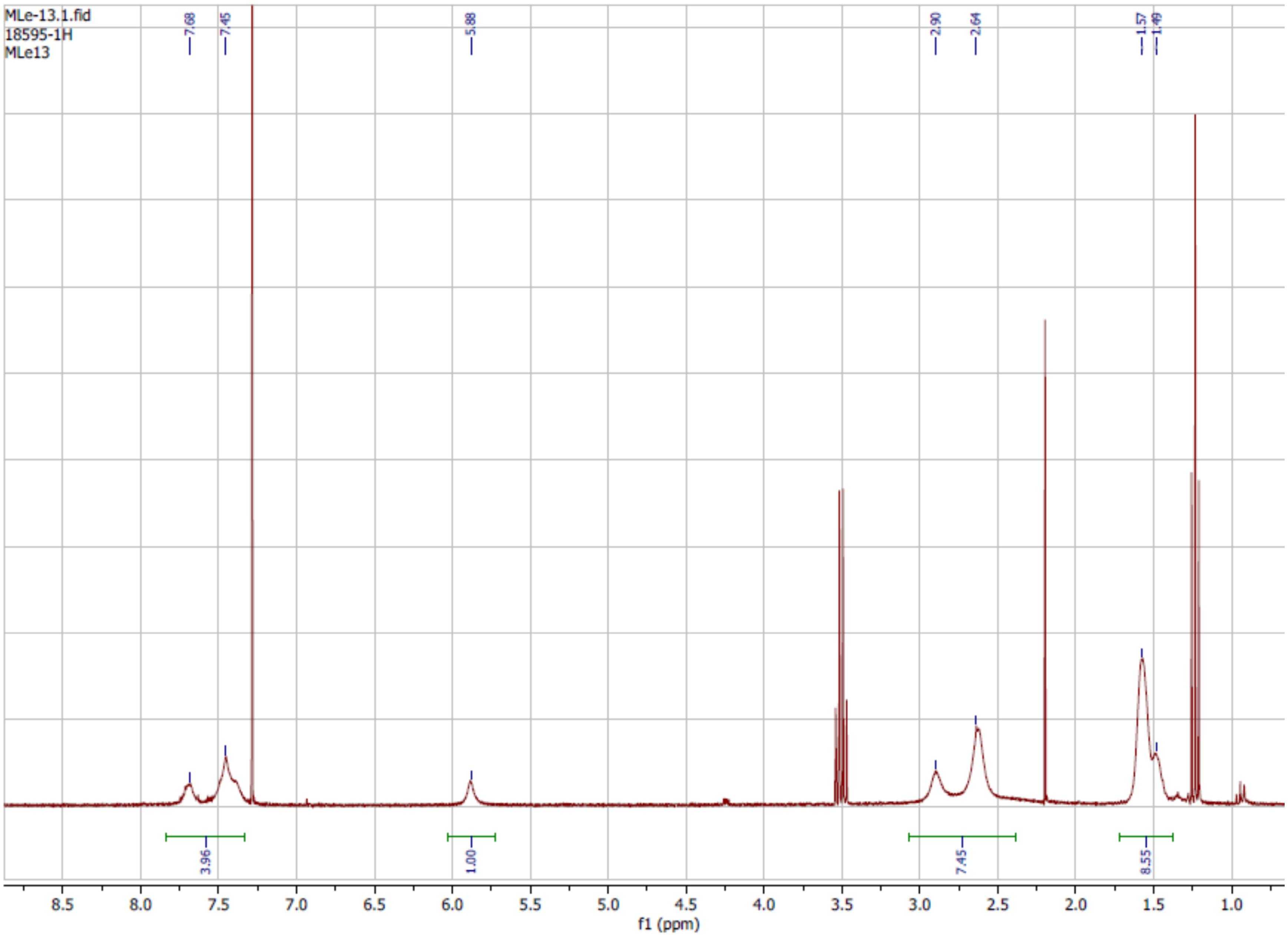

Fig. S.26. *[1] H NMR spectrum of compound **9** in CDCl3.*

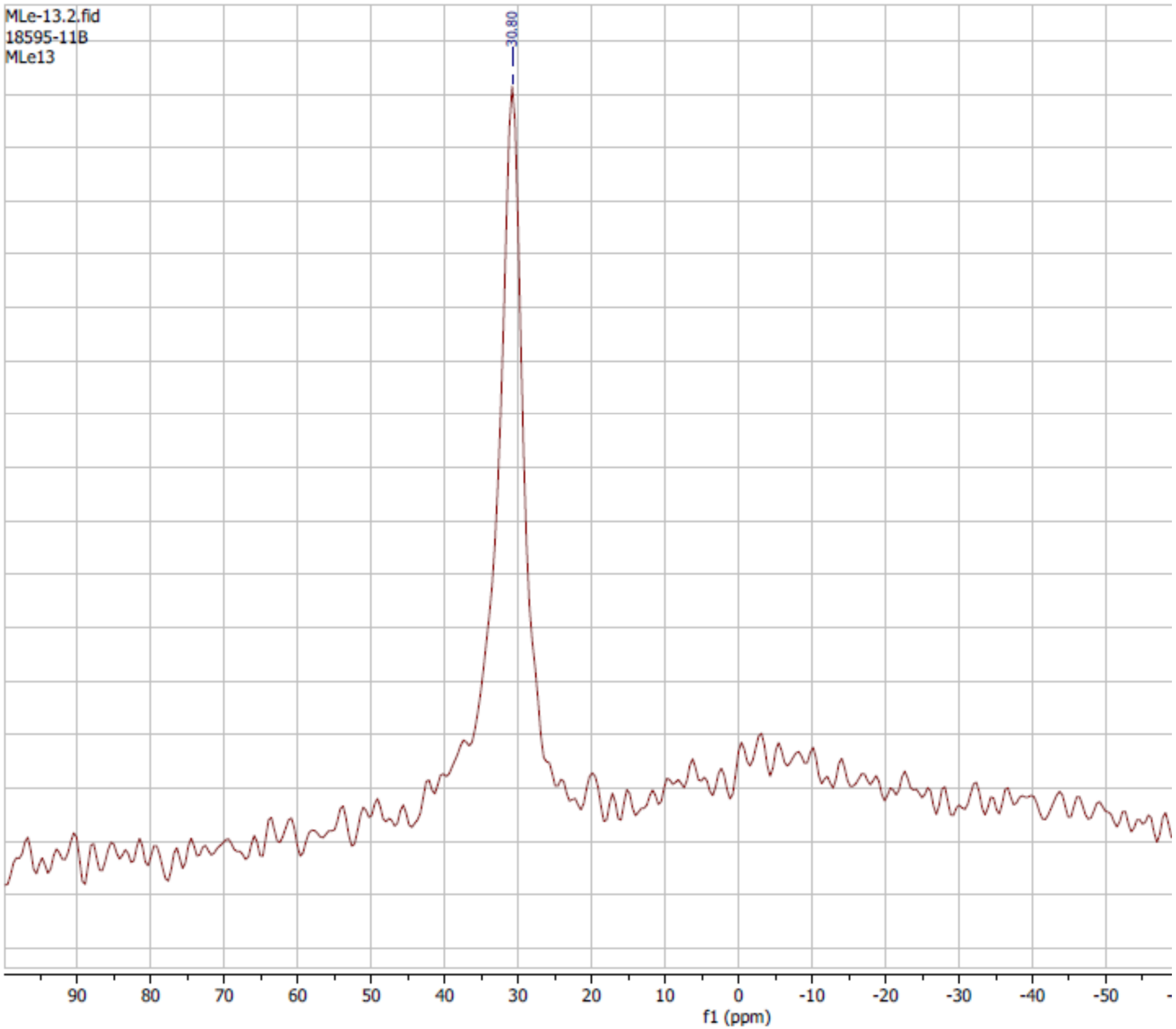

Fig S.27. *[11] B NMR spectrum of compound* ***9*** *in* $CDCl_3$

NMR spectra of compound **10 (MWI-1c)**

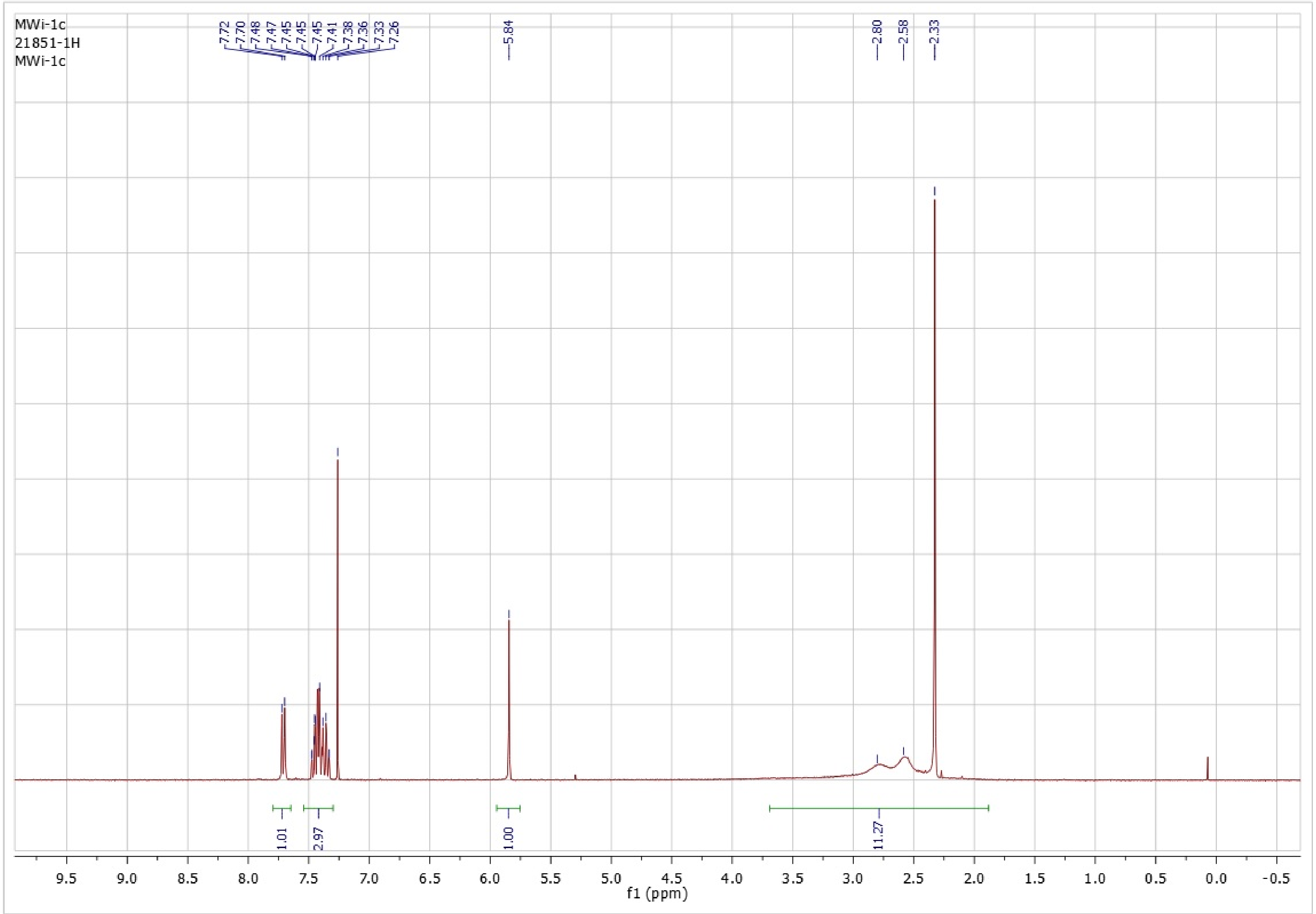

Fig. S.28. *^{1}H NMR spectrum of compound **10** in $CDCl_3$.*

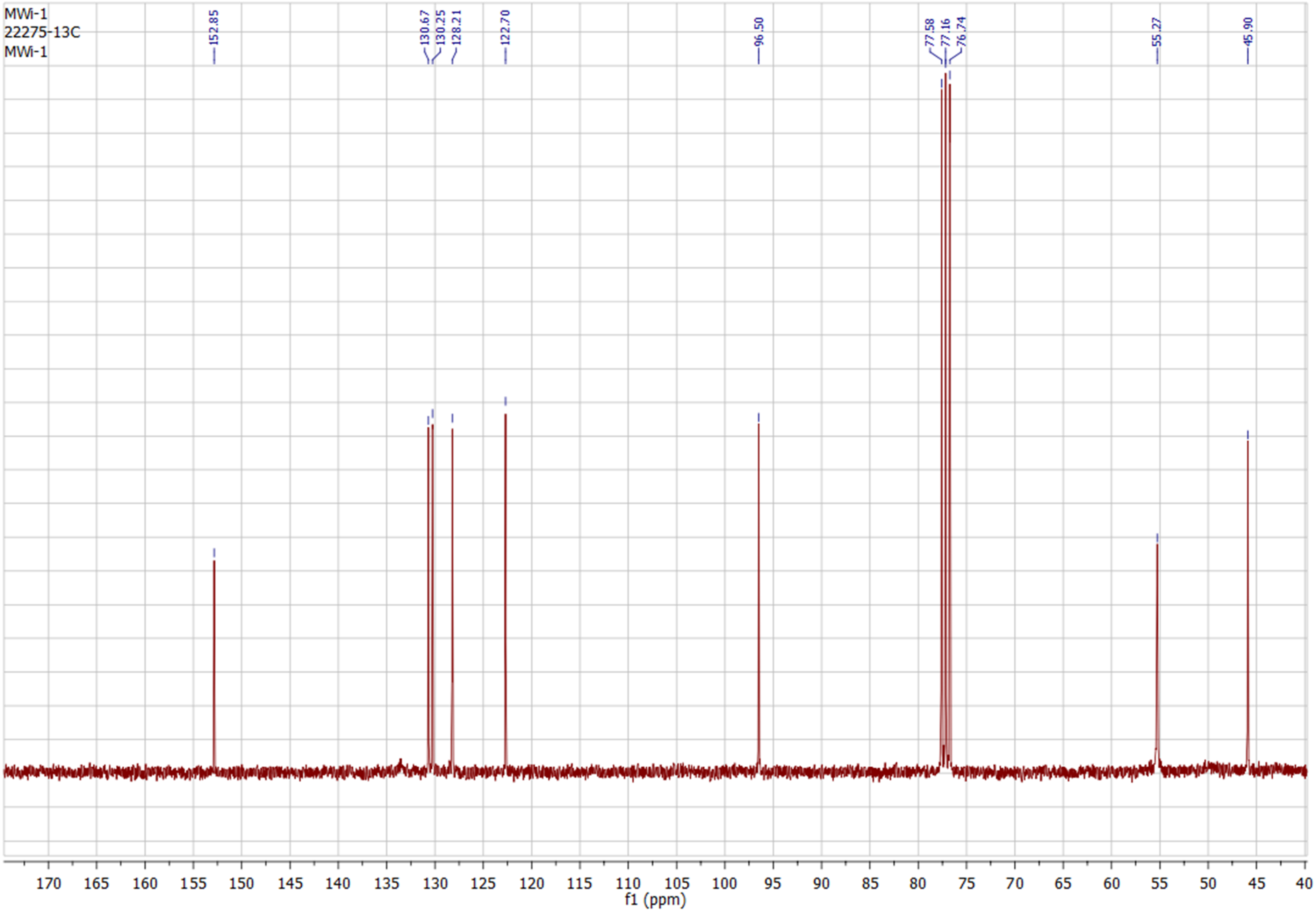

Fig. S.29. *^{13}C NMR spectrum of compound **10** in $CDCl_3$.*

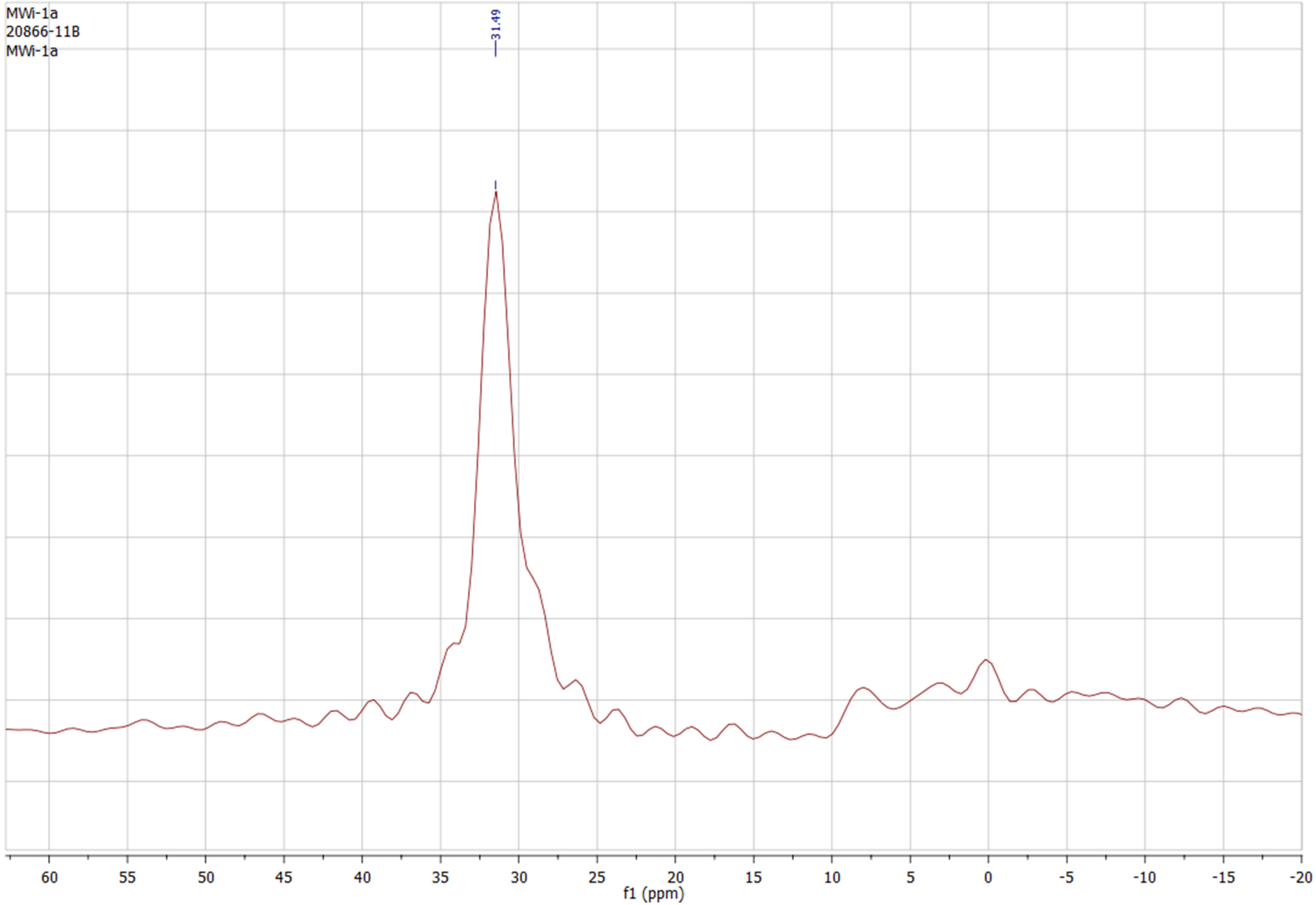

Fig. S.30. *^{11}B NMR spectrum of compound* ***10*** *in CDCl3.*

NMR spectra of compound **11**

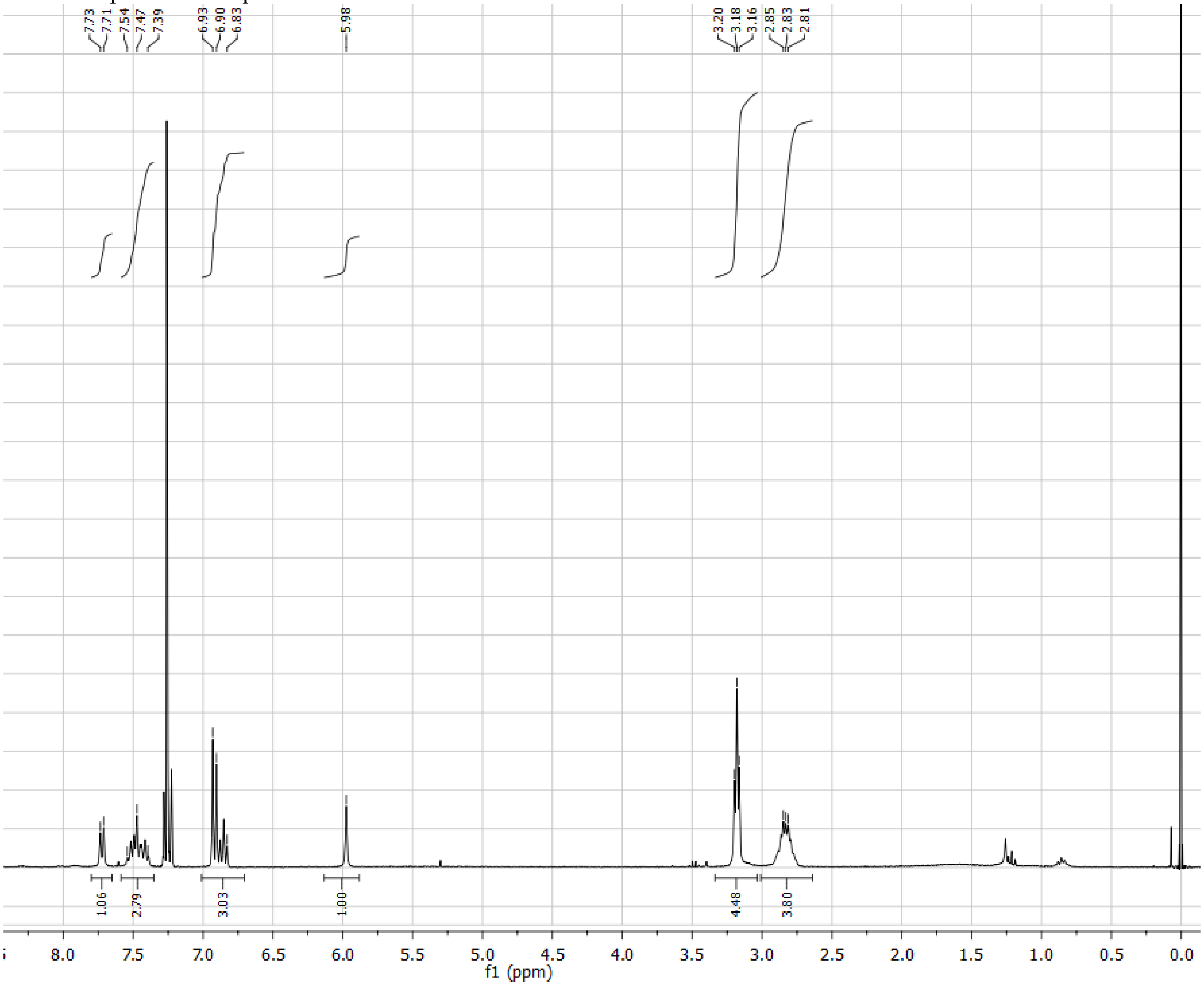

Fig. S.31. *^{1}H NMR spectrum of compound **11** in $CDCl_3$.*

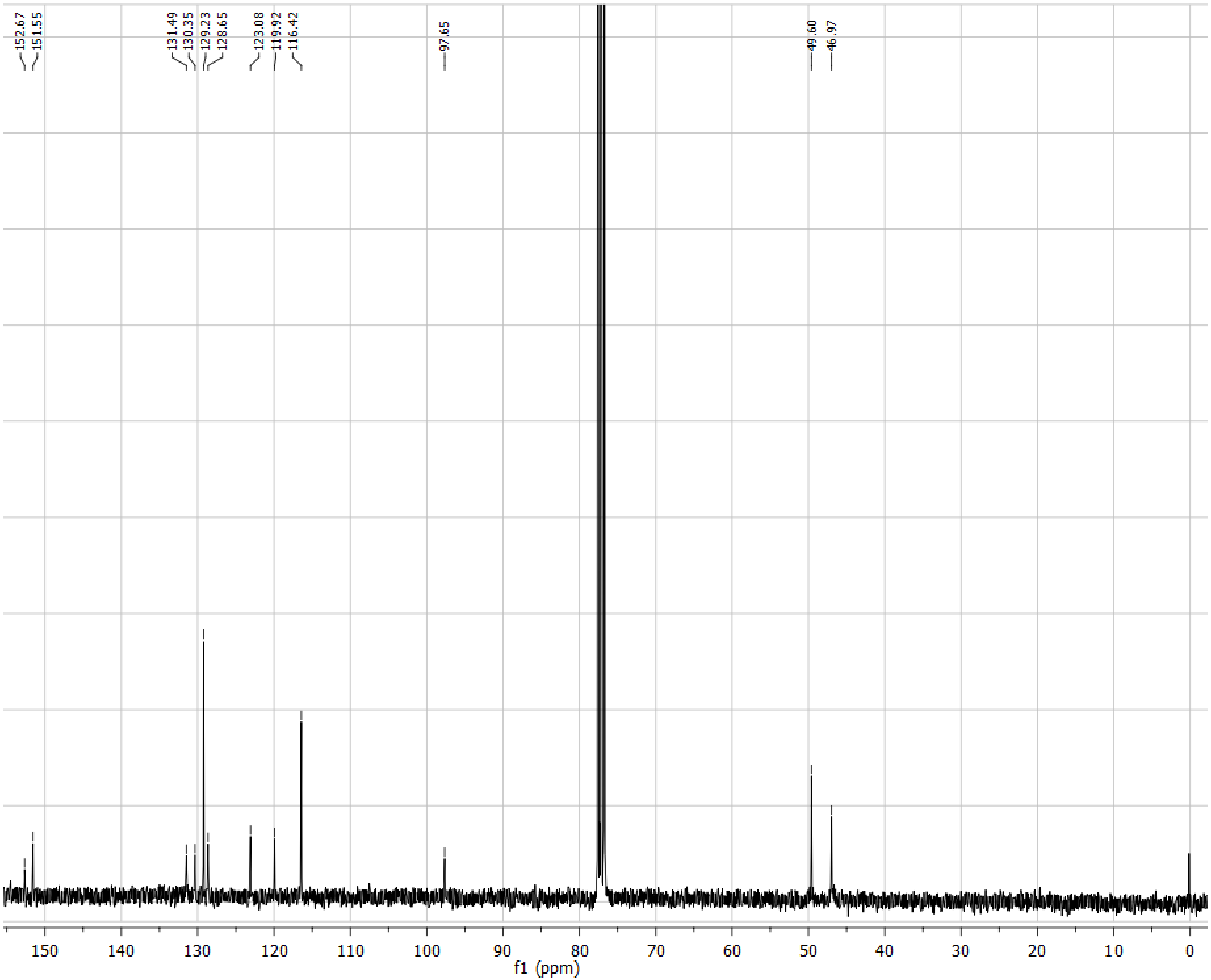

Fig. S.32. *^{13}C NMR spectrum of compound* ***11*** *in* $CDCl_3$**.**

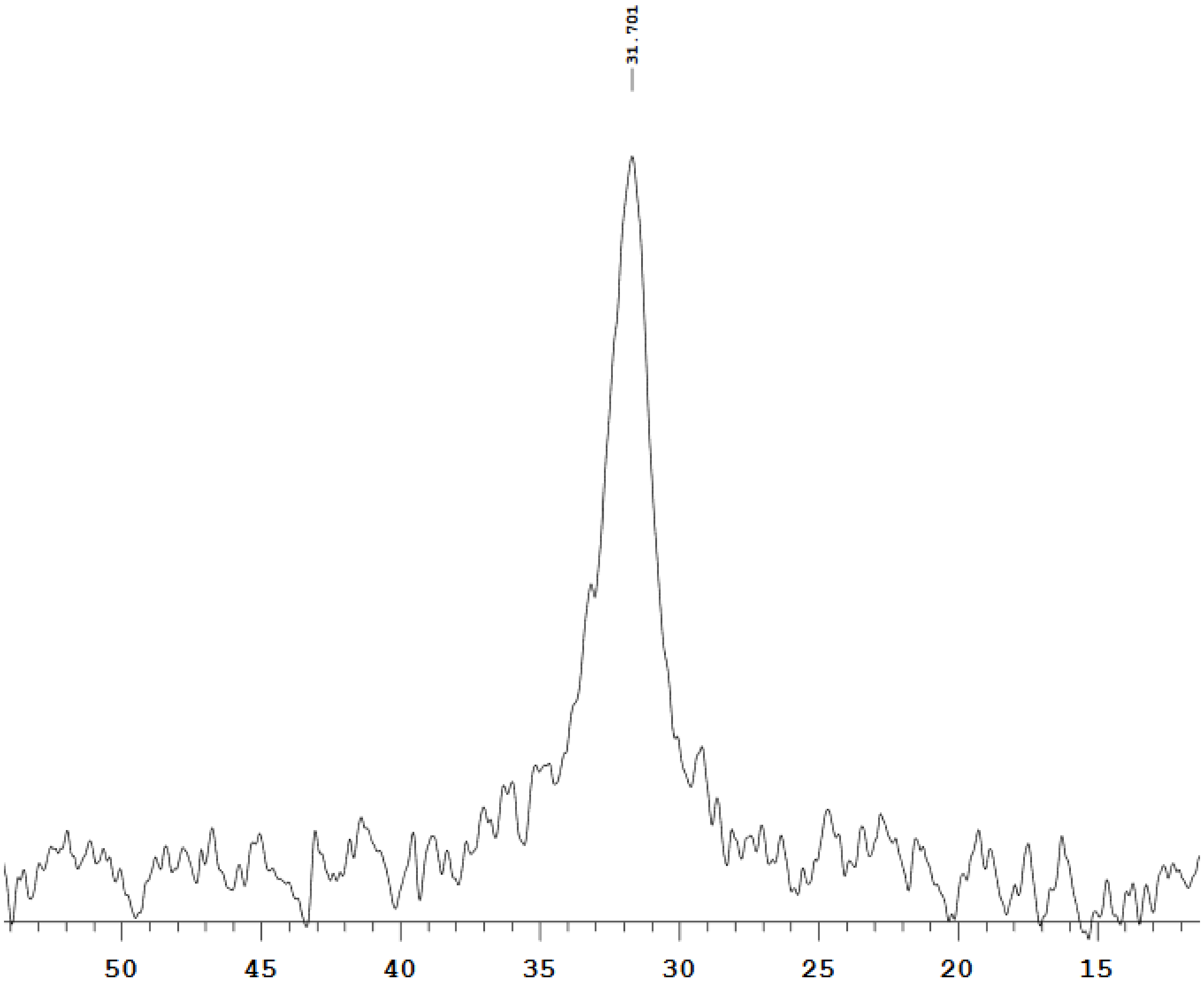

Fig. S.33. *^{11}B NMR spectrum of compound **11** in $CDCl_3$.*

NMR spectra of compound **14**

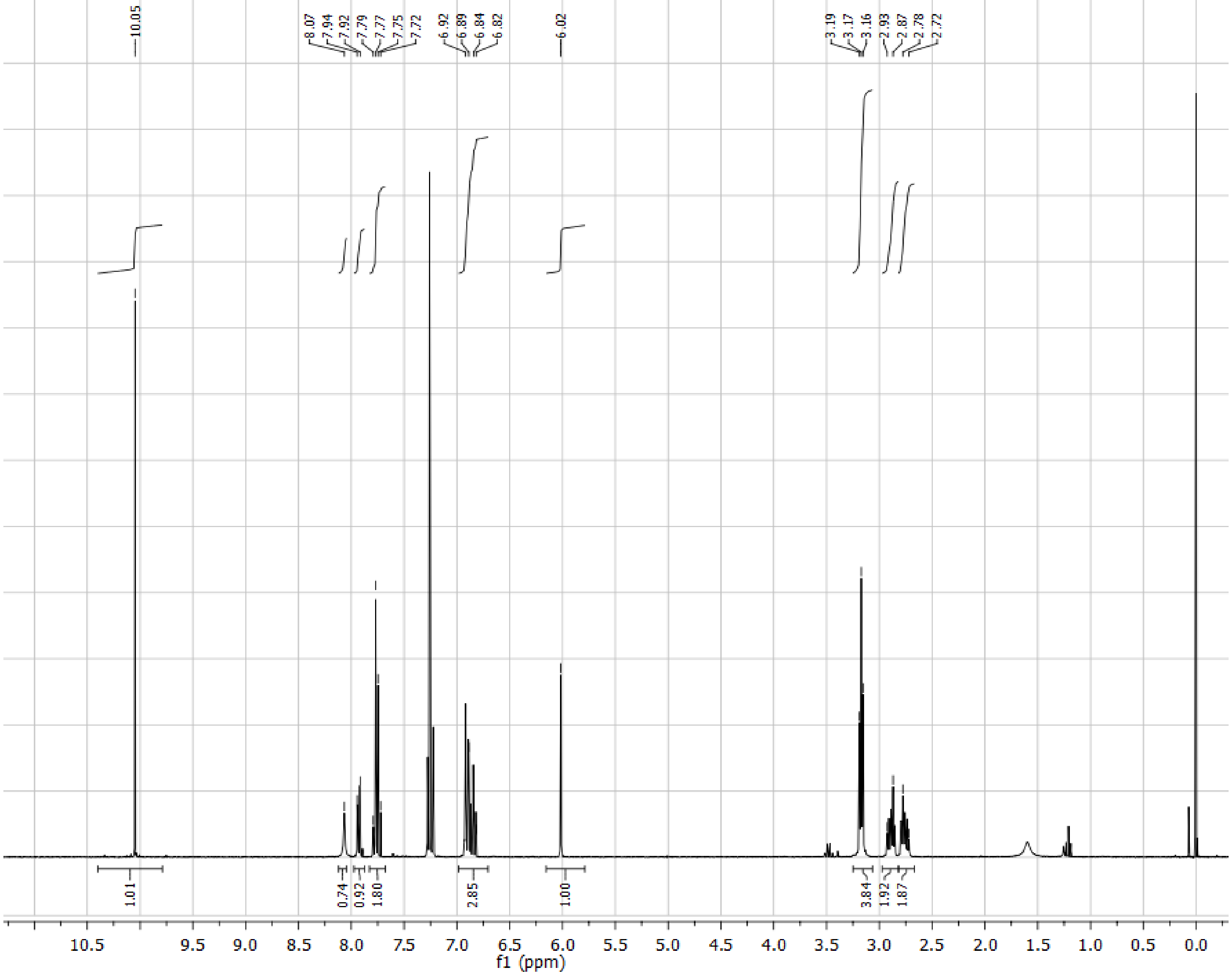

Fig. S.34. *^{1}H NMR spectrum of compound* ***14*** *in* $CDCl_3$.

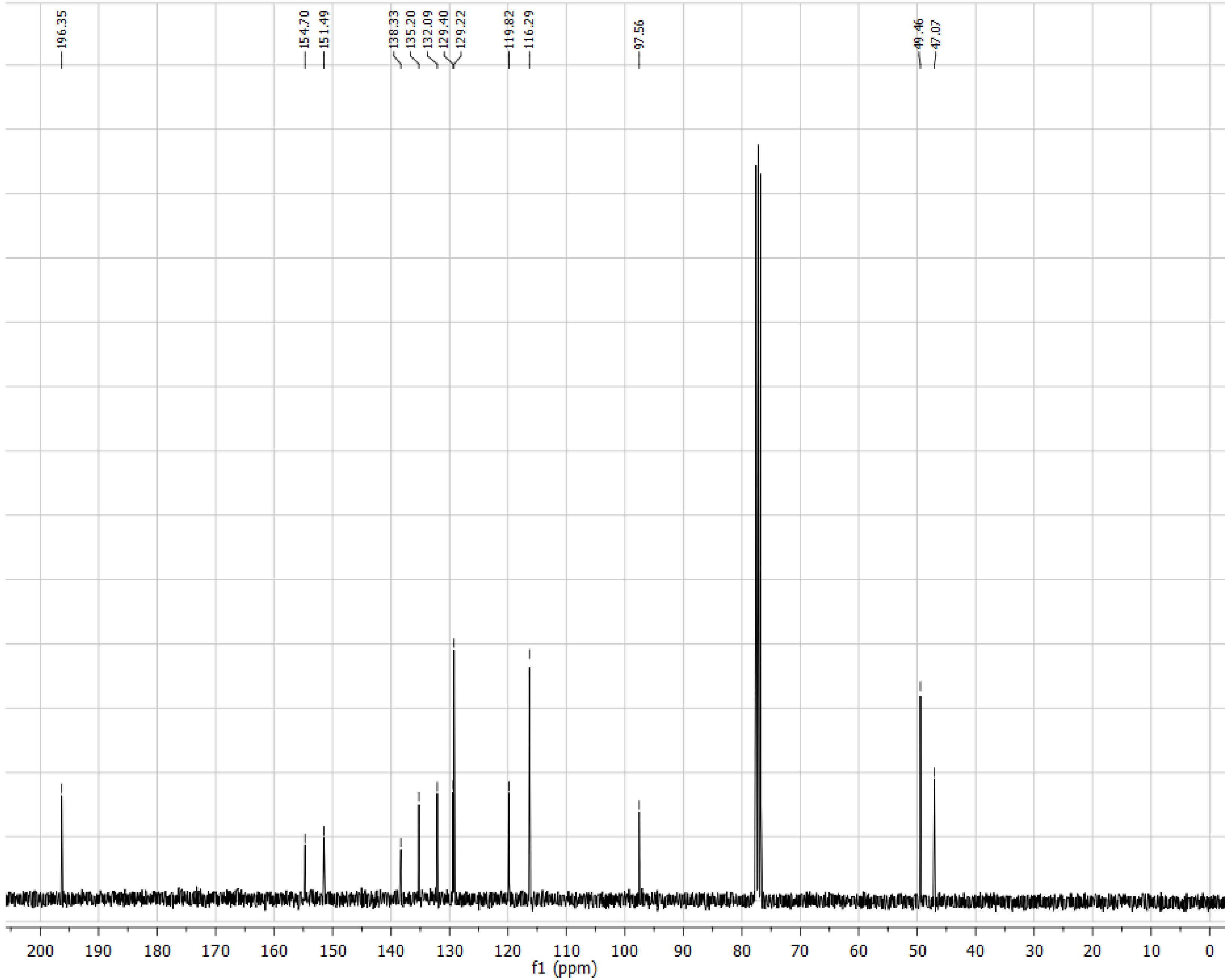

Fig. S.35. *[13] C NMR spectrum of compound* ***14*** *in $CDCl_3$.*

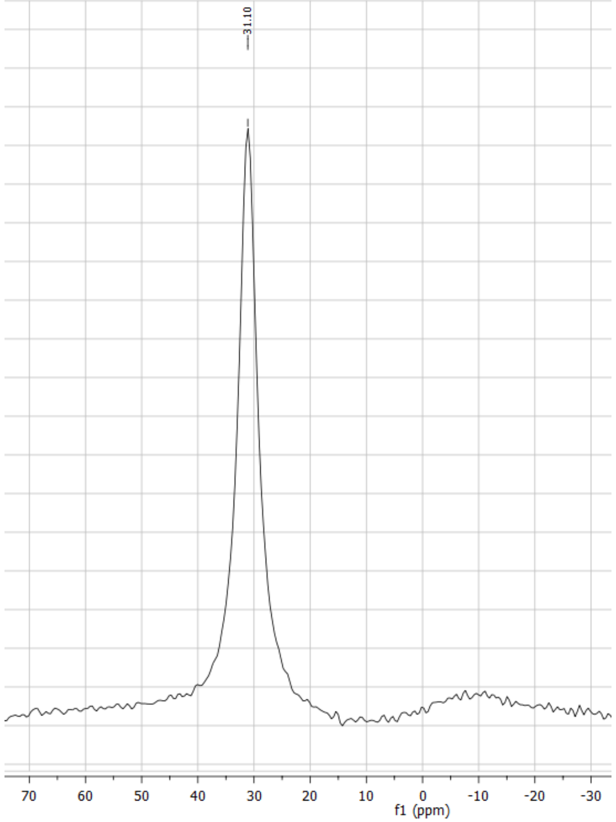

Fig. S.36. *[11] B NMR spectrum of compound **14** in CDCl₃.*

NMR spectra of compound **15**

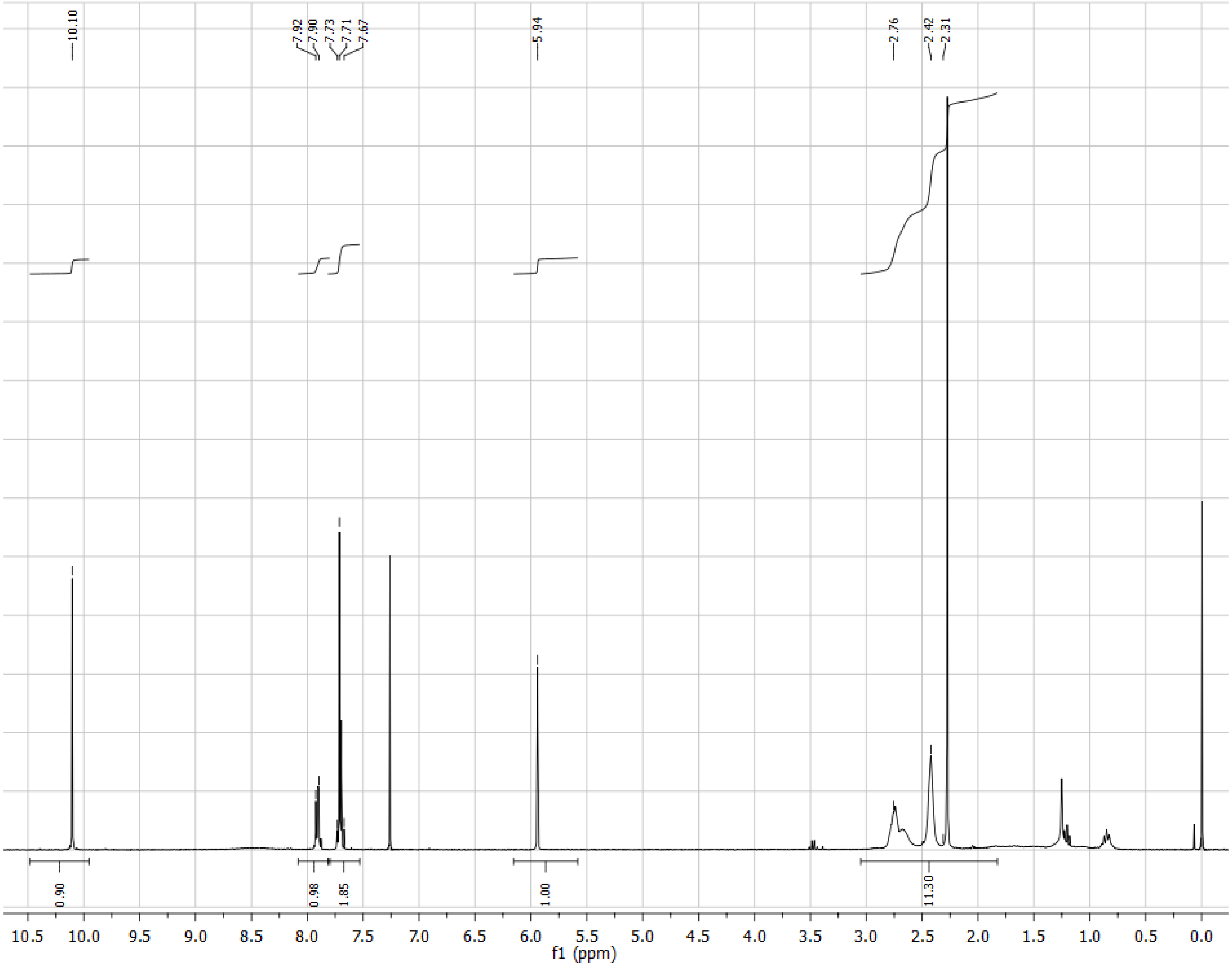

Fig. S.37. *^{1}H NMR spectrum of compound **15** in $CDCl_3$.*

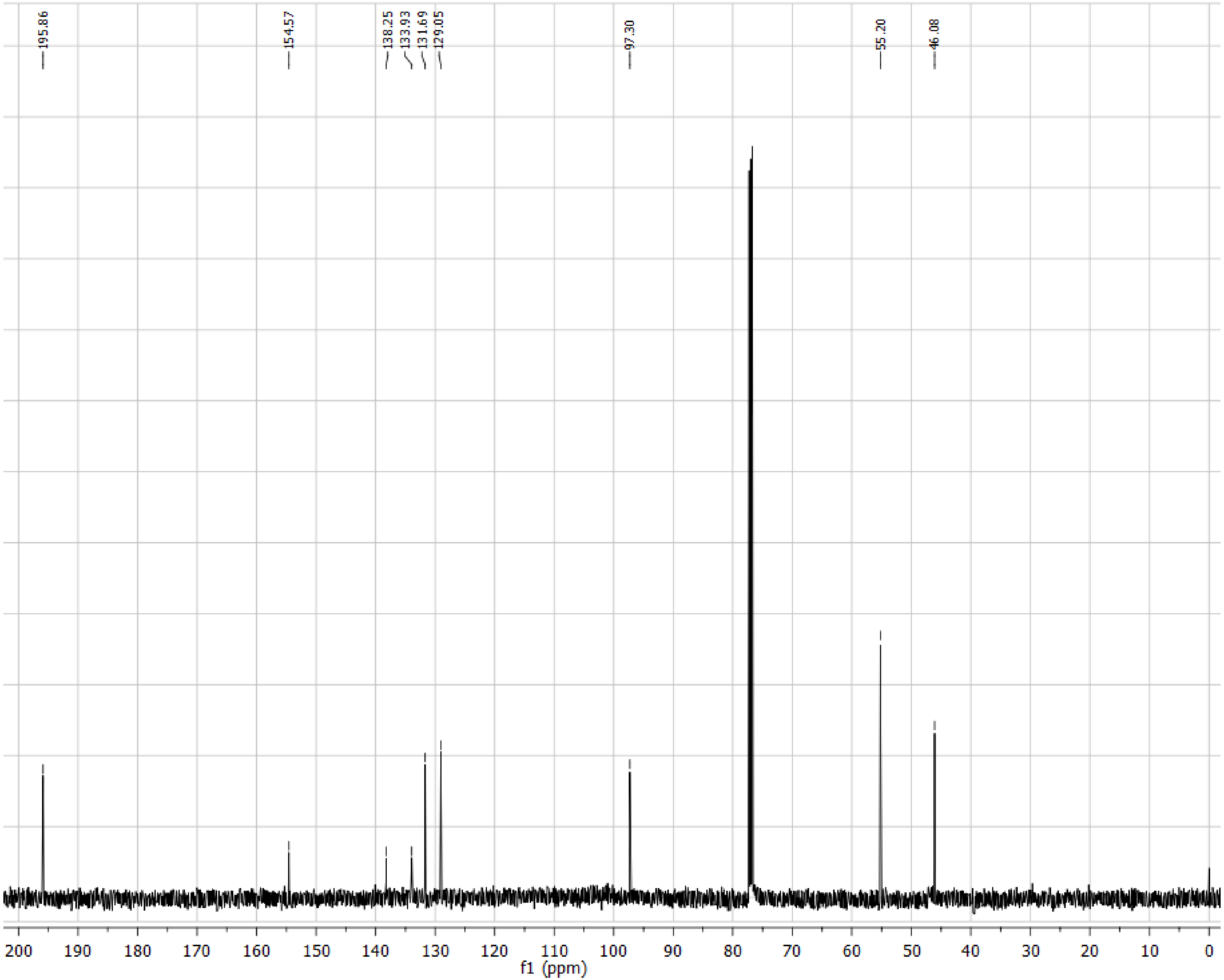

Fig. S.38. *[13] C NMR spectrum of compound **15** in $CDCl_3$.*

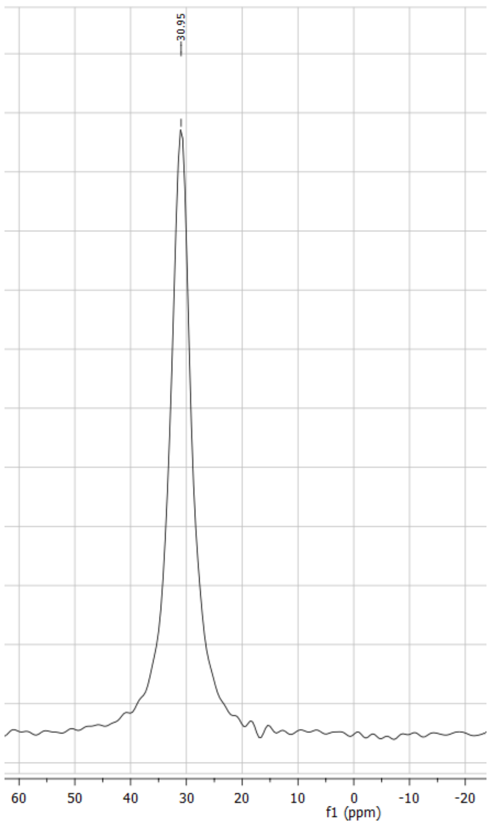

Fig. S.39. *[11] B NMR spectrum of compound **15** in* $CDCl_3$.
